# CIRP attenuates acute kidney injury after hypothermic cardiovascular surgery by inhibiting PHD3/HIF-1α-mediated ROS-TGF-β1/p38 MAPK activation and mitochondrial apoptotic pathways

**DOI:** 10.1186/s10020-023-00655-0

**Published:** 2023-05-01

**Authors:** Peiyao Zhang, Liting Bai, Yuanyuan Tong, Shengwen Guo, Wenlong Lu, Yue Yuan, Wenting Wang, Yu Jin, Peng Gao, Jinping Liu

**Affiliations:** 1 State Key Laboratory of Cardiovascular Disease, National Center for Cardiovascular Diseases, Fuwai Hospital, Chinese Academy of Medical Sciences and Peking Union Medical College, Beijing, 102308 China; 2 Department of Cardiopulmonary Bypass, Fuwai Hospital, Chinese Academy of Medical Sciences and Peking Union Medical College, No.167, North Lishi Road, Xicheng District, Beijing, 100037 China; 3 Department of Anesthesiology, Beijing Friendship Hospital, Capital Medical University, Beijing, 100050 China; 4 Department of Anesthesiology, Beijing Tiantan Hospital, Capital Medical University, Beijing, 100070 China; 5 Department of Anesthesiology, Xiamen Cardiovascular Hospital, Xiamen University, Xiamen, Fujian 361000 China; 6 Department of Endocrinology, Drum Tower Hospital affiliated to Nanjing University Medical School, Branch of National Clinical Research Centre for Metabolic Diseases, Nanjing, Jiangsu 210008 China

**Keywords:** Cold inducible RNA-binding protein, Ischemia–reperfusion, Renoprotection, Deep hypothermic circulatory arrest, Apoptosis, Reactive oxygen species, Hypoxia-inducible factor 1α, Enarodustat

## Abstract

**Background:**

The ischemia–reperfusion (IR) environment during deep hypothermic circulatory arrest (DHCA) cardiovascular surgery is a major cause of acute kidney injury (AKI), which lacks preventive measure and treatment. It was reported that cold inducible RNA-binding protein (CIRP) can be induced under hypoxic and hypothermic stress and may have a protective effect on multiple organs. The purpose of this study was to investigate whether CIRP could exert renoprotective effect during hypothermic IR and the potential mechanisms.

**Methods:**

Utilizing RNA-sequencing, we compared the differences in gene expression between *Cirp* knockout rats and wild-type rats after DHCA and screened the possible mechanisms. Then, we established the hypothermic oxygen–glucose deprivation (OGD) model using HK-2 cells transfected with siRNA to verify the downstream pathways and explore potential pharmacological approach. The effects of CIRP and enarodustat (JTZ-951) on renal IR injury (IRI) were investigated in vivo and in vitro using multiple levels of pathological and molecular biological experiments.

**Results:**

We discovered that *Cirp* knockout significantly upregulated rat *Phd3* expression, which is the key regulator of HIF-1α, thereby inhibiting HIF-1α after DHCA. In addition, deletion of *Cirp* in rat model promoted apoptosis and aggravated renal injury by reactive oxygen species (ROS) accumulation and significant activation of the TGF-β1/p38 MAPK inflammatory pathway. Then, based on the HK-2 cell model of hypothermic OGD, we found that *CIRP* silencing significantly stimulated the expression of the TGF-β1/p38 MAPK inflammatory pathway by activating the PHD3/HIF-1α axis, and induced more severe apoptosis through the mitochondrial cytochrome c-Apaf-1-caspase 9 and FADD-caspase 8 death receptor pathways compared with untransfected cells. However, silencing *PHD3* remarkably activated the expression of HIF-1α and alleviated the apoptosis of HK-2 cells in hypothermic OGD. On this basis, by pretreating HK-2 and rats with enarodustat, a novel HIF-1α stabilizer, we found that enarodustat significantly mitigated renal cellular apoptosis under hypothermic IR and reversed the aggravated IRI induced by CIRP defect, both in vitro and in vivo.

**Conclusion:**

Our findings indicated that CIRP may confer renoprotection against hypothermic IRI by suppressing PHD3/HIF-1α-mediated apoptosis. PHD3 inhibitors and HIF-1α stabilizers may have clinical value in renal IRI.

**Supplementary Information:**

The online version contains supplementary material available at 10.1186/s10020-023-00655-0.

## Background

Despite advancements in surgical techniques and antegrade cerebral prefusion (ACP) strategies, deep hypothermic circulatory arrest (DHCA) remains largely indispensable in certain cardiovascular procedures including aortic arch repair and complicated congenital cardiac surgery (Vekstein et al. 2021; Wagner et al. 2019; Gil-Ruiz Gil-Esparza et al. 2014; Yan et al. 2013). Although deep hypothermia during circulatory arrest improves the tolerance of brain to ischemia by decreasing the metabolic rate, DHCA has been identified as a risk factor for acute kidney injury (AKI) (Wagner et al. 2019; Gil-Ruiz Gil-Esparza 2014; Roh et al. 2012), which may render postoperative morbidity and mortality (Thomas et al. 2015; Lameire et al. 2013). Kidneys are highly sensitive to oxygen supply. Extensive renal tubule injury and renal function impairment have been observed when exposed to hypoxia (Liu et al. 2018; Legrand et al. 2008), and a systemic ischemia–reperfusion (IR) environment during DHCA would undoubtedly aggravate this process. Considering the limited therapeutic measures for AKI, it is imperative to explore potential renoprotective pathways for prevention and targeted treatment.

Cold inducible RNA-binding protein (CIRP) is a highly sequence-homologous, low-level and ubiquitously expressed nuclear protein in mammals that was initially discovered in mechanistic research on cold stress adaptation (Hiroyuki Nishiyama 1997a, b; Zhu et al. 2016; Zhong and Huang 2017; Qiang et al. 2013). Under the conditions of hypothermia, hypoxia, ultraviolet (UV) radiation, shock and other cellular stresses (Gotic et al. 2016; Liao et al. 2017; Liu et al. 2010; Wellmann et al. 2004), CIRP is inclined to be induced promptly to stabilize specific mRNAs. CIRP coordinates mRNA translational reprogramming by selectively binding to the 3’-untranslated region of transcripts, which facilitates cellular adaptation to uncomfortable environments (Hiroyuki Nishiyama 1997b; Zhong and Huang 2017; Liao et al. 2017). Accumulating data demonstrate that CIRP may exert protective effects on multiple organs during IR (Liu et al. 2019, 2020; Yu et al. 2018; Li et al. 2019; McGinn et al. 2018), and the available in vivo renal phenotypic evidence suggests that CIRP may play an anti-apoptotic role during hypothermic cardioplegic arrest (Yu et al. 2018). However, the underlying molecular mechanism remains unclear.

Therefore, we established rat and human renal cell models to simulate clinical hypothermic IR and inactivated human *CIRP* or rat *Cirp* by genetic approaches. Through RNA-sequencing and molecular biological analyses, we explored the specific role and underlying mechanisms of CIRP in DHCA to offer implications for therapeutic targets to restrain injury in kidneys and other organs.

## Methods

All animal protocols were approved by the Fuwai Hospital Animal Care and Use Committee.

### Generation of *Cirp*^*−/−*^ rats and rat model of DHCA

*Cirp* knockout (*Cirp*^*−/−*^) rats with a Sprague–Dawley (SD) background were acquired from Professor Hao Zhang’s laboratory and were generated by transcription activator-like effector nuclease-based genome editing techniques targeting exon 3 of the *Cirp* gene, as previously reported (Liu 2019;). Other male SD rats (13–15 weeks, 350–400 g) were obtained from Huafukang Laboratory Animal Corporation (Beijing, China).

Rat models of DHCA were established to simulate clinical practice as previously described (Additional file 1: Figure S1, Additional file 2: Figure S2) (Liu et al. 2019, 2020). Briefly, rats were monitored via three-lead electrocardiograph (ECG) under general anesthesia with 1.5% sevoflurane by endotracheal intubation and peritoneal injection with 3 μg/kg fentanyl. All rats were mechanically ventilated at 60 breaths/min with a tidal volume of 6–10 mL/kg. After skin and surgical preparation, the left femoral artery was cannulated with a 24-gauge catheter for circulatory monitoring and blood-gas analysis. A 20-gauge catheter was inserted into the caudal artery as the arterial infusion line, and the homemade multiorifice drainage catheter was cannulated into the right superior vena cava through the right external jugular vein as the venous drainage line of the cardiopulmonary bypass (CPB) circuit. The rectal temperature during CPB was monitored by a temperature sensor. The CPB circuit consisted of a homemade venous reservoir, a heat exchanger (Kewei, Beijing, China), a rodent membrane oxygenator (Kewei, Beijing, China), a mini roller pump (Stöckert, Munich, Germany), a heat exchange water cabinet (Maquet, Solna, Sweden) and connecting silicon tubes. The entire circuit was primed with 1 mL unfractionated heparin (250 IU/kg), 11 mL 6% hydroxyethyl starch (Hospira, New York, USA), 0.5 mL 5% sodium bicarbonate (Kelun, Chengdu, China). After heparinization, CPB was initiated with a flow rate of over 120 mL/kg/min. As the core temperature cooled to 18 °C within 30 min, the flow rate was gradually decreased to 0, and DHCA was maintained for 1 h at 18 °C based on ECG monitoring. Then, CPB was restarted, and the rats were rewarmed for 1 h to 34 °C by means of a heating blanket. Subsequently, CPB was weaned when circulatory indices were stable. After 30 min of observation, the rats were euthanized and sampled. Samples of arterial blood-gas analysis were taken at 5 time points: before CPB initiation (T0), 5 min after cooling (T1), 30 min after cooling (T2), 5 min after rewarming (T3), and at the weaning of CPB (T4). Rats in the sham group received anesthesia and endotracheal intubation only. Only the right kidney was harvested for subsequent examination to avoid the influence of blood drawn from the left femoral artery.

### RNA-sequencing

The right kidney tissues in 3 biological replicates of each treatment were collected for RNA-sequencing. RNA extraction, RNA quality examination, library preparation for transcriptome sequencing, clustering, sequencing, differentially expressed gene (DEG) analysis, assembly, and function enrichment analysis including gene ontology (GO) and Kyoto Encyclopedia of Genes and Genomes (KEGG) analysis, were performed as previously reported (Liu et al. 2019).

### Histological analysis

Hematoxylin–eosin (H&E) staining was performed, and tissue damage (tubular epithelial swelling, brush border loss, vacuolar degeneration, desquamation, cast formation and necrotic tubules) was assessed in line with Leelahavaniehkul’s method (Leelahavanichkul et al. 2008). Briefly, five randomly selected fields of each sample at × 200 magnification were assessed by a blinded observer. The degree of kidney damage was scored by the percentage of impaired tubules: 0, normal; 1, < 25%; 2, 25–50%; 3, 50–75%; 4, 75–100%.

### Biochemical index measurement

Serum creatinine (SCr)(RN-04. 0201A, Weigao, Shandong, China), blood urea nitrogen (BUN) (100020070, Biosino, Beijing, China) and uric acid levels (100020110, Biosino, Beijing, China) of each rat were measured using the Labospect 008 AS (Hitachi, Tokyo, Japan) and matched assay kits following the manufacturer’s protocol.

### Enzyme-linked immunosorbent assay

Serum interleukin-1β (IL-1β)(F15810), interleukin-6 (IL-6)(F15870), tumor necrosis factor-α (TNF-α)(F16960), cystatin C (F15270), renal tissue kidney injury molecule-1 (KIM-1)(F16101), neutrophil gelatinase-associated lipocalin (NGAL)(F16344), postoperative urine insulin-like growth factor binding protein-7 (IGFBP-7) (FS13100) and tissue inhibitor of metalloproteinase-2 (TIMP-2) (F16940) levels of each rat were detected using corresponding commercial enzyme-linked immunosorbent assay (ELISA) kits (Westang, Shanghai, China). In addition, the levels of IL-1β (F01220), IL-6 (F01310) and TNF-α (F02810) in the lysate and culture medium of HK-2 cells were quantified using ELISA kits (Westang, Shanghai, China) according to the manufacturer’s instructions.

### Real-time quantitative polymerase chain reaction

Total RNA was extracted using TRIzol reagent (Invitrogen, Carlsbad, USA). Reverse transcription was performed using oligo (dT) primers and the PrimeScript RT reagent kit (Takara, Shiga, Japan). Quantitative reverse transcription-polymerase chain reaction (qRT–PCR) was conducted using the SYBR green-based kit (Thermo Fisher, Waltham, USA) and ABI 7500 system (Applied Biosystems, Foster City, USA) according to the manufacturer’s instructions. Specific gene primers are shown in Additional file 8: Table S1.

### Western blot

Total protein and cytoplasmic protein without mitochondrial protein was extracted from rat kidney and HK-2 cells using RIPA buffer (Beyotime, Shanghai, China) and a tissue/cell mitochondria isolation kit (Beyotime, Shanghai, China). Briefly, protein samples were resolved by sodium dodecyl sulfate–polyacrylamide gel electrophoresis (SDS–PAGE) and transferred to a polyvinylidene difluoride (PVDF) membrane (Millipore, Billerica, USA). The membranes were blocked with 5% skim milk for 2 h and incubated with primary antibodies overnight at 4 °C. Then, they were incubated with horseradish peroxidase (HRP)-conjugated secondary antibodies for 1 h at room temperature (RT). The membranes were developed using a chemiluminescence imaging system (Tanon, Shanghai, China). Relative expression was quantified as the ratio to the expression of β-actin protein. Protein expression was quantified using ImageJ software (Version 1.53, National Institutes of Health, USA). Specific primary and secondary antibodies with dilutions are presented in Additional file 8: Table S2.

### Immunohistochemistry and immunofluorescence

For immunohistochemistry, deparaffinized tissue section (5 μm) were blocked with 5% goat serum and incubated with primary antibodies overnight at 4 °C. Then, sections were incubated with fluorochrome-conjugated secondary antibodies at RT for 45 min. Images were captured by a panoramic scanning system (3DHISTECH, Budapest, Hungary).

For immunofluorescence, sections of rat kidney tissue and HK-2 cells were incubated with primary antibodies overnight at 4 °C. After being washed in phosphate-buffered saline (PBS) for 3 times, sections were incubated with Alexa Fluor 488-conjugated or 647-conjugated goat anti-mouse or anti-rabbit antibodies (Abcam, Cambridge, USA) for 45 min at RT. 4, 6-diamidino-2 phenyindole dilactate (DAPI, Beyotime, Shanghai, China) was used for nuclear staining. Images were captured using an SP8 laser-scanning confocal microscope (Leica, Wetzlar, Germany) at × 400 magnification. Specific primary and secondary antibodies with dilutions are presented in Additional file 8: Table S2.

Quantitative measurement was determined by the percentage of immune-positive cells in 5 randomly selected microscopic vision fields (Nezic et al. 2020).

### Transmission electron microscopy

Renal tissues (1 mm^3^) were successively fixed with 2.5% glutaraldehyde and 1% osmium tetroxide. Thereafter, tissues were dehydrated in ethanol, embedded in epoxy and cut into ultrathin slices of 80 nm with an ultramicrotome (Leica, Wetzlar Germany). Then, slices were stained with uranyl acetate and lead citrate. A transmission electron microscopy (TEM) system (JEM-1400plus, JEOL, Tokyo, Japan) was adopted for image collection.

### Cell culture and hypothermic oxygen–glucose deprivation model

The human proximal tubular epithelial cell line HK-2 (Procell, Wuhan, China) was cultured in Roswell Park Memorial Institute 1640 medium (RPMI-1640, Sigma–Aldrich, St. Louis, USA) containing 10% fetal bovine serum (FBS, Sigma–Aldrich, St. Louis, USA), 100 U/mL penicillin and 100 μg/mL streptomycin in a normoxic (21% O_2_) incubator with 5% CO_2_ and 74% N_2_ at 37 °C.

For the hypothermic oxygen–glucose deprivation (OGD) model, HK-2 cells were first cultured in glucose-free and serum-free RPMI-1640 medium and placed into a poikilothermic hypoxic incubator with 1% O_2_, 5% CO_2_ and 94% N_2_ at 18 °C for 2, 4, 6 or 8 h (to determine the optimal protocol according to the expression of hypoxia-associated protein). After that, the medium was replaced with complete RPMI-1640 medium containing 10% FBS, and the cells were transferred into a normoxic incubator for 30 min. HK-2 cells cultured in complete medium containing 10% FBS and placed in a normoxic incubator at 37 °C were classified as the control group.

### RNA interference

Human *CIRP* and prolyl hydroxylase 3 (*PHD3*) small interfering RNA (siRNA) as well as negative control siRNA (siNC) were designed and synthesized by Genepharma Pharmaceutical Technologies Inc. (Shanghai, China). The *CIRP* siRNA (siCIRP) duplex consisted of sense 5′-GGCCAUGAAUGGGAAGUCUTT-3′ and antisense 5′-AGACUUCCCAUUCAUGGCCTT-3′. *PHD3* siRNA (siPHD3) duplex consisted of sense 5′-GCAUCUACUAUCUGAACAATT-3′ and antisense 5′-UUGUUCAGAUAGUAGAUGCTT-3′.

Cells were transfected with siRNA using Lipofectamine RNAiMAX Transfection Reagent (Thermo Fisher, Waltham, USA) and Opti-MEM I medium (Thermo Fisher, Waltham, USA) according to the manufacturer’s instructions for 48 h in RPMI-1640 medium containing 3% FBS without antibiotics.

### Flow cytometry

HK-2 cells were double-stained with annexin V and propidium iodide (PI) by flow cytometry to detect cell apoptosis using the fluorescein isothiocyanate (FITC) annexin V apoptosis detection kit I (BD, Franklin Lakes, USA). The results were analyzed with FlowJo software (Version 10.4, Treestar, Ashland, USA).

### TdT-mediated dUTP nick end labeling assay

Sections of paraffin-embedded rat renal tissues and 4% paraformaldehyde fixed HK-2 cells were stained by a TdT-mediated dUTP nick end labeling (TUNEL) kit (Beyotime, Shanghai, China). DAPI (Beyotime, Shanghai, China) was used for nuclear staining. Photos were captured under an SP8 laser-scanning confocal microscope (Leica, Wetzlar, Germany) at × 400 magnification. The apoptotic degree was quantified as the percentage of TUNEL-positive cells in 5 randomly selected fields (Nezic et al. 2020).

### Reactive oxygen species assay

The content of reactive oxygen species (ROS) in rat kidney and HK-2 cells was detected by dichlorodihydrofluorescein diacetate (DCFH-DA) using a tissue ROS assay kit (Bestbio, Shanghai, China) and a cellular ROS assay kit (Beyotime, Shanghai, China). According to the manufacturer’s protocol, ROS in tissues were examined by a fluorescence microplate reader (Tecan, Männedorf, Switzerland), and ROS in cells were detected by inverted fluorescence microscopy (Leica, Wetzlar, Germany) at × 200 magnification and a fluorescence microplate reader.

### Cell vitality assay

The cell vitality of HK-2 cells was detected using a cell counting kit-8 (CCK-8, Beyotime, Shanghai, China) according to the manufacturer’s instructions. The absorbance was measured at 450 nm using a microplate reader (Tecan, Männedorf, Switzerland).

### Mitochondrial membrane potential assay

Mitochondrial membrane potential (MMP) was measured using a commercial kit (Beyotime, Shanghai, China) according to the manufacturer’s protocol. The fluorescence intensity of MMP in HK-2 cells was measured using a fluorescence microplate reader (Tecan, Männedorf, Switzerland).

### ATP content assay

The ATP content of HK-2 cells was detected using the enhanced ATP assay kit (Beyotime, Shanghai, China) according to the manufacturer’s instructions. Briefly, HK-2 cells in each group were lysed on ice and centrifuged at 12,000 for 5 min. 20 μl of supernatant was collected and mixed with 180 μl working solution for 4 min. The relative optical unit (RLU) value was measured by a fluorescence microplate reader (Tecan, Männedorf, Switzerland) to calculate the ATP concentration.

### Statistical analysis

All data are presented as the mean ± standard deviation (SD). Statistical analysis was performed using SPSS 25.0 software (SPSS, Chicago, USA) and figures were drawn using GraphPad Prism 8.0 software (GraphPad, San Diego, USA). Differences between two groups were compared using Student’s t test, and comparisons among no less than three groups were examined by one-way analysis of variance (ANOVA) followed by the Tukey test. All experiments were conducted at least in triplicate. Two-tailed P < 0.05 was considered statistically significant.

## Results

### *Cirp* knockout exacerbated renal injury after DHCA

**Fig. 1:**
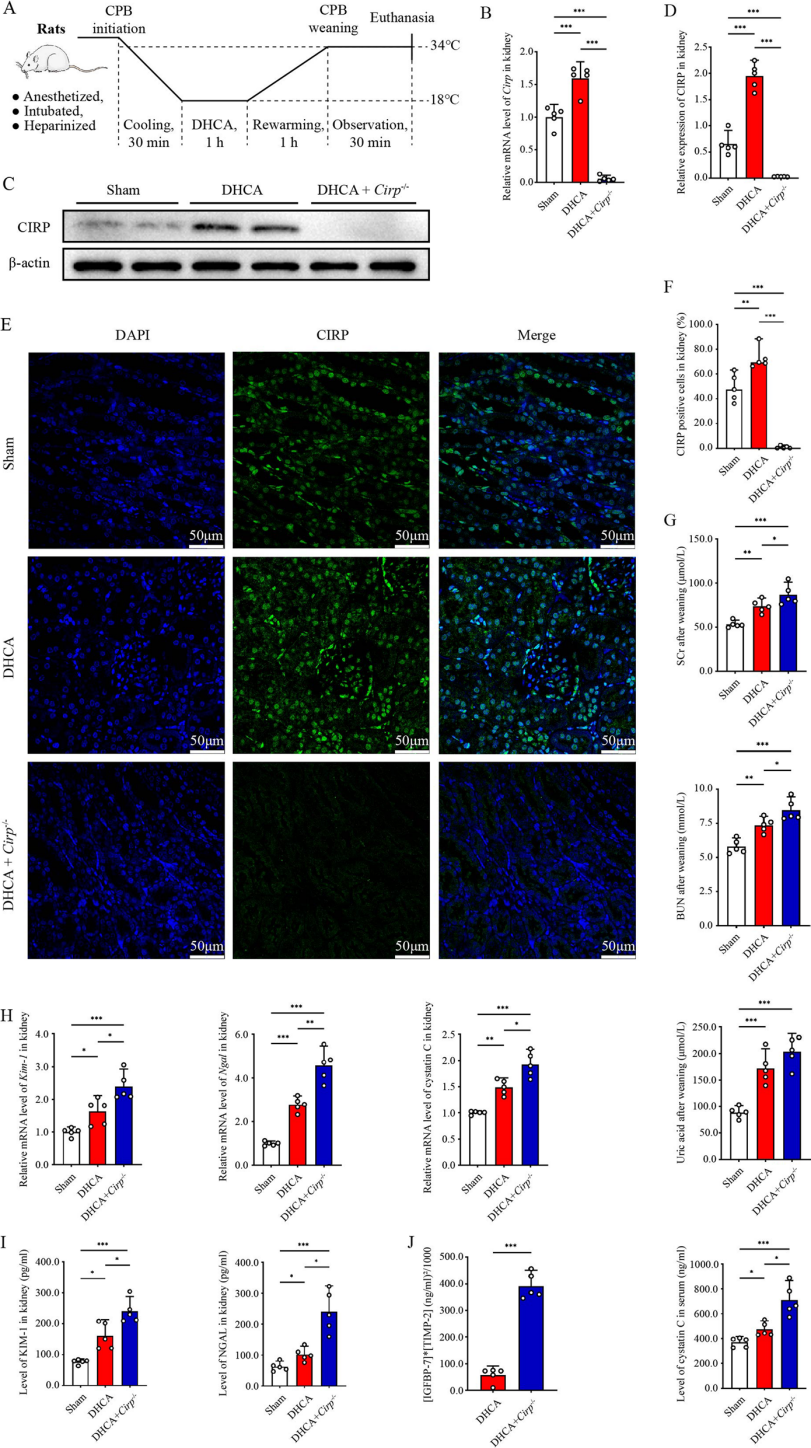
CIRP was induced by DHCA and *Cirp* knockout impaired renal function after DHCA. **A** Schematic illustration of experimental time and temperature flow of rat DHCA model. **B** Level of *Cirp/*β-actin mRNA in kidneys from the sham, DHCA and DHCA + *Cirp*^*−/−*^ groups (n = 5 per group). **C** Representative image of western blot analysis of CIRP in renal tissues from the three groups. **D** The ratio of CIRP to β-actin by western blot analysis. **E** Immunofluorescence staining of kidneys from three groups (original magnification, × 400). **F** The CIRP-positive cells were quantified by the percentage in 5 randomly selected microscopic vision fields. (G) Postoperative serum creatinine (SCr), blood urea nitrogen (BUN), uric acid and cystatin C in the three groups. **H** Levels of *Kim-1/*β-actin, *Ngal/*β-actin *and* cystatin C*/*β-actin mRNA in kidneys from the three groups. **I** ELISA analyses of renal KIM-1 and NGAL in the three groups. **J** Postoperative urine (IGFBP-7) × (TIMP-2) in the DHCA and the DHCA + *Cirp*^*−/−*^ groups (n = 5 per group). Statistical differences between two groups were compared using Student’s t test, and comparisons among no less than three groups were examined by one-way analysis of variance (ANOVA) followed by the Tukey test. *P < 0.05, **P < 0.01, ***P < 0.001

Experimental rats were randomly assigned to 3 groups (n = 5 in each group): sham group, DHCA group and DHCA + *Cirp*^*−/−*^ group (*Cirp*^*−/−*^ group). All rats underwent DHCA operation according to the standard protocol as Fig. 1A illustrated. Perioperative physiological index monitoring and blood-gas analysis confirmed that the rat DHCA model was successfully established (Additional file 8: Table S3). All rats survived until euthanasia 30 min after weaning of cardiopulmonary bypass (CPB). Baseline characteristics were comparable among the three groups. qRT–PCR and western blot analysis revealed that *Cirp* was significantly induced in the DHCA group compared with the sham group, whereas *Cirp* was inhibited by over 80% in the *Cirp*^*−/−*^ group, which attested to the efficacy of *Cirp* gene knockout (Fig. 1B–D). Immunofluorescence staining confirmed *Cirp* gene deletion in *Cirp*^*−/−*^ rats and the enhanced expression of CIRP protein under hypothermic IR conditions (Fig. 1E, F), as reported in previous studies (Hiroyuki Nishiyama 1997; De Leeuw et al. 2007; Zhang et al. 2015).

**Fig. 2:**
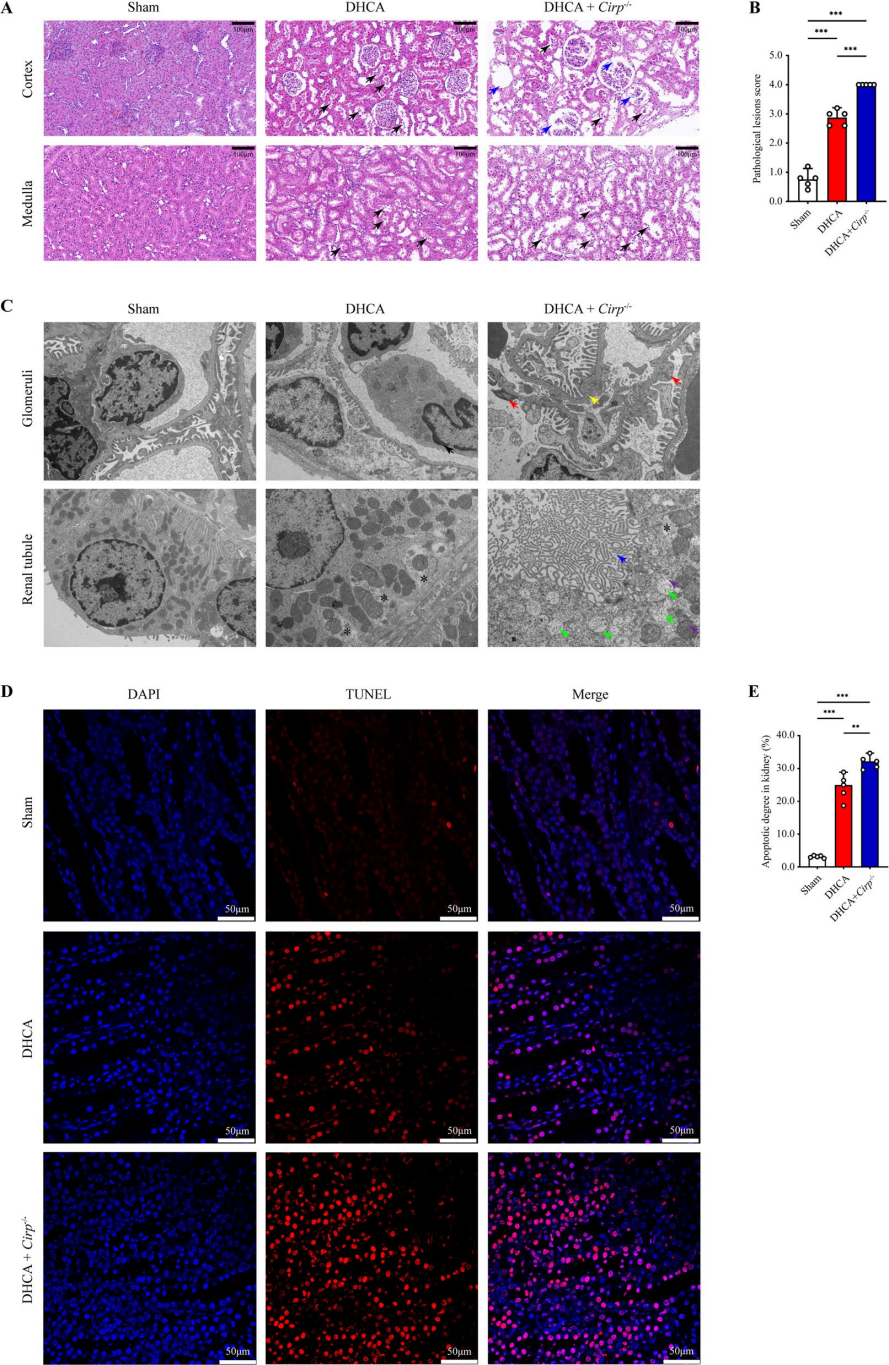
*Cirp* deficiency intensified DHCA-induced renal injury. **A** Representative H&E staining images of the renal cortex and medulla in the sham, DHCA and DHCA + *Cirp*^*−/−*^ groups (original magnification, × 200). Black arrows, exfoliation of renal tubular epithelial cells and cellular cast formation. Blue arrows, glomerular pyknosis and disintegration. **B** Pathological lesion score of renal samples from three groups. **C** Representative TEM images of glomeruli and renal tubule from three groups (original magnification, × 2500). Black arrows, karyopyknosis. Yellow arrows, thickening of the glomerular endothelial basement membrane. Red arrows, partial fusion of foot process. Green arrows, mitochondrial edema, mitochondrial electron density decrease and mitochondrial crest loss. Purple arrows, mitochondrial rupture. Blue arrows, brush border structure disorder. Asterisk, apoptotic body. **D** TUNEL staining of kidneys from the three groups (original magnification, × 400). **E** Apoptotic degree was counted as the percentage of TUNEL signal in 5 randomly selected fields. Statistical significance was examined by one-way analysis of variance (ANOVA) followed by the Tukey test. **P < 0.01, ***P < 0.001

The DHCA group and *Cirp*^*−/−*^ group suffered more severe renal injury than the sham group (Figs. 1, 2). When exposed to hypothermic IR, *Cirp* knockout compromised renal function of rats, resulting in significant increase in SCr, BUN, uric acid and serum cystatin C (Fig. 1G). Simultaneously, the relative mRNA levels of renal injury markers, tissue kidney injury molecule-1 (*Kim-1*), neutrophil gelatinase-associated lipocalin (*Ngal*) and cystatin C increased significantly in *Cirp*^*−/−*^ rats that underwent DHCA (Fig. 1H). Consistently, rats in the *Cirp*^*−/−*^ group had higher protein levels of tissue KIM-1 and NGAL than those in the DHCA group (Fig. 1I). Notably, urine insulin-like growth factor binding protein-7 (IGFBP-7) × tissue inhibitor of metalloproteinase-2 (TIMP-2), a novel indicator of acute kidney injury (Vijayan et al. 2016; Kashani et al. 2013; Wang et al. 2017;), was significantly higher in the *Cirp*^*−/−*^ group than in the DHCA group (Fig. 1J).

As H&E staining showed, *Cirp*^*−/−*^ rats had aggravated tissue damage and higher pathological lesion scores than wild-type rats after DHCA (Fig. 2A, B). Then, TEM was used to evaluate the effect of *Cirp* knockout on renal ultrastructure. Figure 2C demonstrates that DHCA caused obvious damage to renal cells, mainly manifesting as early apoptotic signs, including thickening of the glomerular endothelial basement membrane, brush border structure disorder, karyopyknosis, mitochondrial edema and apoptotic body formation. *Cirp* knockout further aggravated cell damage characterizing by mitochondrial crest loss, decreased of mitochondrial electron density, mitochondrial disintegration and nuclear rupture. Accordingly, we performed TUNEL staining to assess the apoptosis of rat kidney cells. The results showed that the *Cirp*^*−/−*^ group had a significantly higher apoptotic degree than the DHCA group (24.3% vs. 32.0%, P < 0.05, Fig. 2D, E).

Collectively, these results suggested that *Cirp* knockout exacerbated DHCA-induced renal injury by damaging mitochondria and promoting apoptosis.

### PHD3/HIF-1α axis mediated renal injury aggravated by *Cirp* knockout

**Fig. 3:**
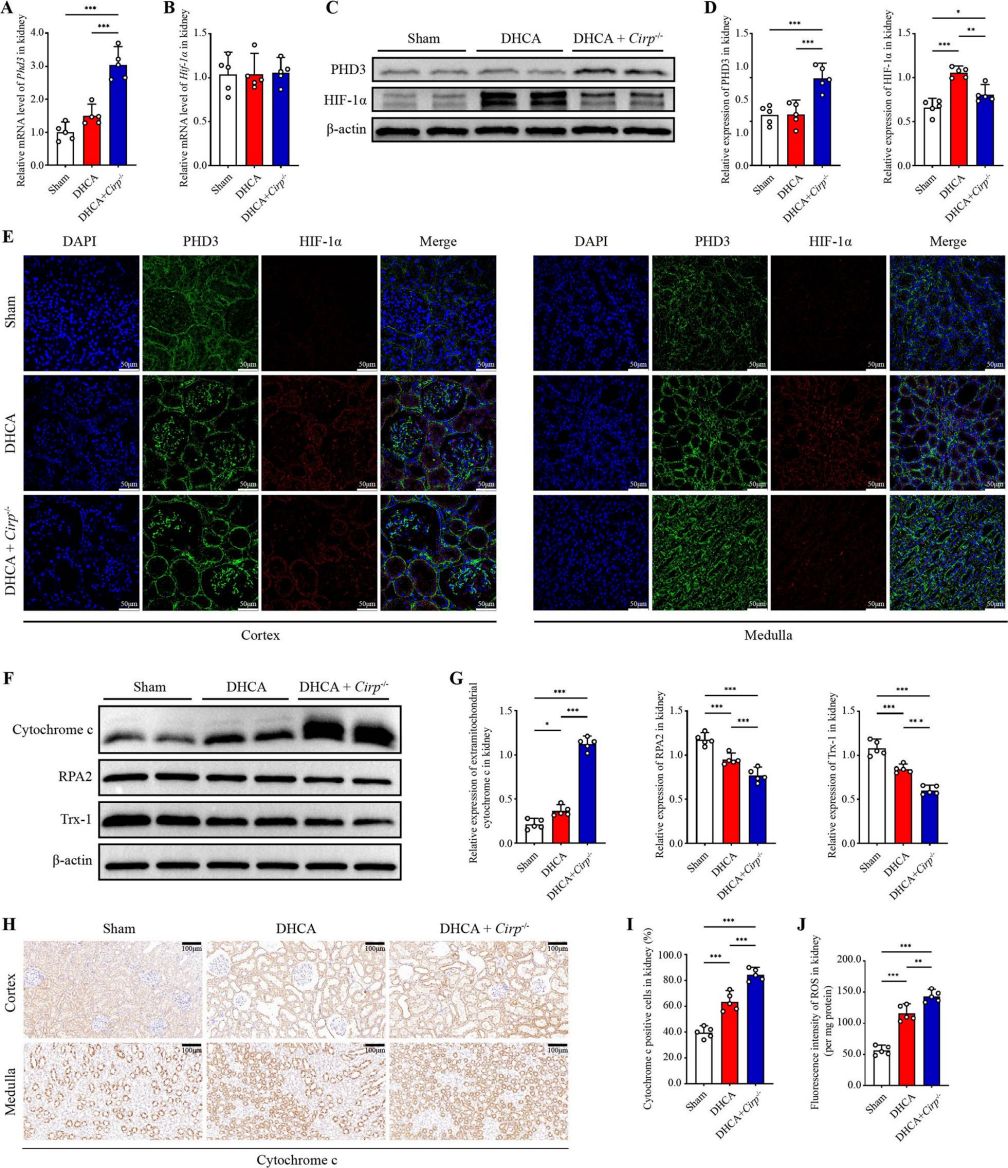
*Cirp* knockout aggravated mitochondrial damage and ROS accumulation via PHD3/HIF-1α axis. **A, B** Levels of *Phd3/*β-actin and *Hif-1α/*β-actin mRNA in kidneys from the sham, DHCA and DHCA + *Cirp*^*−/−*^ groups (n = 5 per group). **C** Representative western blot images of PHD3 and HIF-1α in renal tissues from the three groups. **D** The ratio of PHD3 and HIF-1α to β-actin by western blot analysis. **E** Representative immunofluorescence images of PHD3 and HIF-1α in the renal cortex and medulla from the three groups (original magnification, × 400). **F** Representative images of extramitochondrial cytochrome c, renal RPA2 and Trx-1 in renal tissues from the three groups by western blot analysis. **G** The ratio of extramitochondrial cytochrome c, renal RPA2 and Trx-1 to β-actin by western blot analysis. **H** Representative immunohistochemistry images of cytochrome c in renal cortex and medulla from the three groups (original magnification, × 200). **I** The cytochrome c-positive cells were quantified by the percentage in 5 randomly selected microscopic vision fields. **J** Fluorescence intensity of ROS in kidneys from the three groups (per mg protein). ROS, reactive oxygen species. Statistical significance was examined by one-way analysis of variance (ANOVA) followed by the Tukey test. *P < 0.05, **P < 0.01, ***P < 0.001

To investigate the molecular mechanism underlying renal injury exacerbated by *Cirp* knockout, we conducted RNA-sequencing analysis on rat kidneys. A total of 303 differentially expressed genes (DEGs) were identified, including 190 upregulated genes and 113 downregulated genes between the DHCA group and the *Cirp*^*−/−*^ group [Log_2_(fold change) > 1 or Log_2_(fold change) < − 1, P < 0.05, Additional file 3: Figure S3A]. These genes were functionally clustered GO and KEGG analyses (Additional file 3: Figure S3B-E). Among these genes, the prolyl hydroxylase 3 (*Phd3*) gene was significantly upregulated in the *Cirp*^*−/−*^ group compared to the DHCA group (fold change 3.93, P < 0.05). *PHD3* (also called *EGLN3*) has been widely identified as a key regulator of hypoxia-inducible factor 1α (HIF-1α) (Kaelin and Ratcliffe 2008; Gunaratnam and Bonventre 2009). Proline residues of the HIF-1α subunit are degraded by hydroxylation of PHD3 under normoxic conditions (Lee et al. 2019; Semenza 2019). Accordingly, we further detected the mRNA and protein levels of *Phd3* and *Hif-1α*. The results showed that *Phd3* mRNA and PHD3 protein were increased significantly in the *Cirp*^*−/−*^ group compared with the DHCA group. The protein level of HIF-1α was significantly decreased in the *Cirp*^*−/−*^ groups, although the mRNA level was unchanged (Fig. 3A–D). These results were further verified by immunofluorescence double-staining of PHD3 and HIF-1α (Fig. 3E), suggesting that alteration of HIF-1α protein may involve posttranslational modification.

GO analysis showed that the upregulated genes were enriched in biological terms including “response to oxygen-containing compound”, “anatomical structure development”, “developmental process”, “negative regulation of biological process”, “plasma membrane part”, “sulfur compound binding” and “oxidoreductase activity” (Additional file 3: Figure S3B-D), which was consistent with our findings of a tendency of mitochondrial disintegration, oxidative stress aggravation and apoptosis in the *Cirp*^*−/−*^ group. Correspondingly, we measured the expression of cytochrome c, the classical oxidoreductase (Santucci et al. 2019), and replication protein A2 (RPA2) and thioredoxin 1 (Trx-1), which are involved in the repair of damaged DNA and reactive oxygen species (ROS) quenching, respectively (Givalos et al. 2007; Yang et al. 2006). Notably, we extracted extramitochondrial cytochrome c by isolating mitochondria from the cytoplasm to detect the extent of mitochondrial damage. As Fig. 3F, G shows, extramitochondrial cytochrome c increased significantly after DHCA, and *Cirp* knockout further increased its level (P < 0.05), indicating that *Cirp* knockout exacerbated DHCA-induced mitochondrial damage. Immunohistochemistry indicated that cytochrome c-positive cells were mainly found in tubular cells rather than in glomerular cells (Fig. 3H), indicating more severe injury in the renal medulla. Furthermore, the numbers of tubular cells expressing cytochrome c in the cytoplasm were significantly higher in the *Cirp*^*−/−*^ group than in the DHCA group, as revealed by immunohistochemistry (Fig. 3H, I), suggesting aggravated cell injury and increased release of cytochrome c. Additionally, the expression levels of RPA2 and Trx-1 were statistically higher in the *Cirp*^*−/−*^ group than in the DHCA group (P < 0.05, Fig. 3F, G). Simultaneously, the level of ROS increased significantly in the *Cirp*^*−/−*^ group compared with the DHCA group (Fig. 3J).

Collectively, these data suggested that *Cirp* knockout aggravated renal injury through the PHD3/HIF-1α axis, and the potential mechanism may involve ROS accumulation and the release of cytochrome c from mitochondria to the cytoplasm.

### *Cirp* knockout promoted the activation of the TGF-β1/p38 MAPK inflammatory pathway

**Fig. 4:**
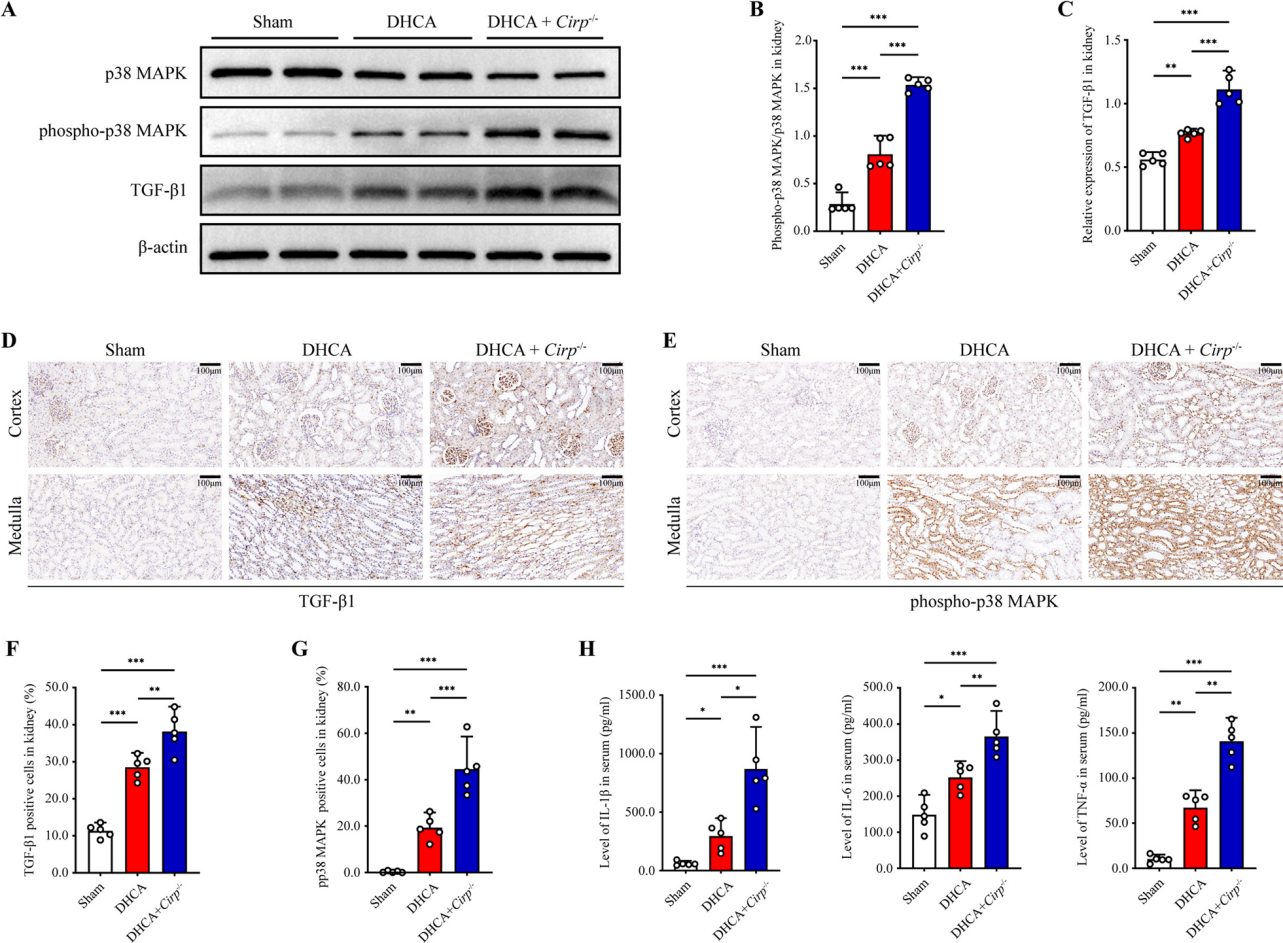
*Cirp*^*−/−*^ activated TGF-β1/p38 MAPK inflammatory pathway. **A** Representative western blot images of p38 MAPK, phospho-p38 (pp38) MAPK and TGF-β1 in kidneys from the sham, DHCA and DHCA + *Cirp*^*−/−*^ groups. **B** The ratio of pp38 MAPK/p38 MAPK by western blot analysis. **C** The ratio of TGF-β1/β-actin by western blot analysis. **D, E** Representative immunohistochemistry images of TGF-β1and pp38 MAPK in the renal cortex and medulla from the three groups (original magnification, × 200). **F, G** The TGF-β1 or pp38 MAPK-positive cells were quantified by the percentage in 5 randomly selected microscopic vision fields. **H** ELISA analyses of serum IL-1β, IL-6 and TNF-α in the three groups. Statistical significance was examined by one-way analysis of variance (ANOVA) followed by the Tukey test. *P < 0.05, **P < 0.01, ***P < 0.001

To further dissect the mechanism underlying the involvement of the CIRP/PHD3/HIF-1α axis and in renal injury, we referred to the results of KEGG analysis (Additional file 3: Figure S3E). By screening for differentially expressed signaling pathways, we discovered that p38 mitogen-activated protein kinase (p38 MAPK) and its upstream target, transforming growth factor-β1 (TGF-β1) (Dubash et al. 2016), were significantly activated in the context of *Cirp* knockout after DHCA. The ratio of phospho-p38 (pp38) MAPK/p38 MAPK and the expression of TGF-β1 were significantly higher in the *Cirp*^*−/−*^ group than in the DHCA group according to western blot analysis (P < 0.05, Fig. 4A–C). Immunohistochemistry revealed similar trends in the expression of activated TGF-β1 and p38 MAPK (pp38 MAPK) among the groups (Fig. 4D–G). P38 MAPK is a classic signaling pathway in the MAPK family that plays an essential role in the process of inflammation, stress response and apoptosis (Yong et al. 2009; Zeyen et al. 2022). To gain insights into whether the activation of TGF-β1/p38 MAPK was involved in the enhanced inflammatory response, we detected the expression of serum inflammatory cytokines. ELISA results showed that the expression levels of interleukin-1β (IL-1β), interleukin-6 (IL-6) and tumor necrosis factor-α (TNF-α) were significantly higher in the DHCA group than in the sham group and were even higher in the *Cirp*^*−/−*^ group (P < 0.05, Fig. 4H).

Collectively, these results suggested that activation of the TGF-β1/p38 MAPK inflammatory pathway might be responsible for enhanced inflammation in the *Cirp*^*−/−*^ group, which may contribute to the aggravation of renal injury.

### *Cirp* knockout may exacerbate renal injury through the mitochondrial apoptotic pathway and death receptor apoptotic pathway

**Fig. 5:**
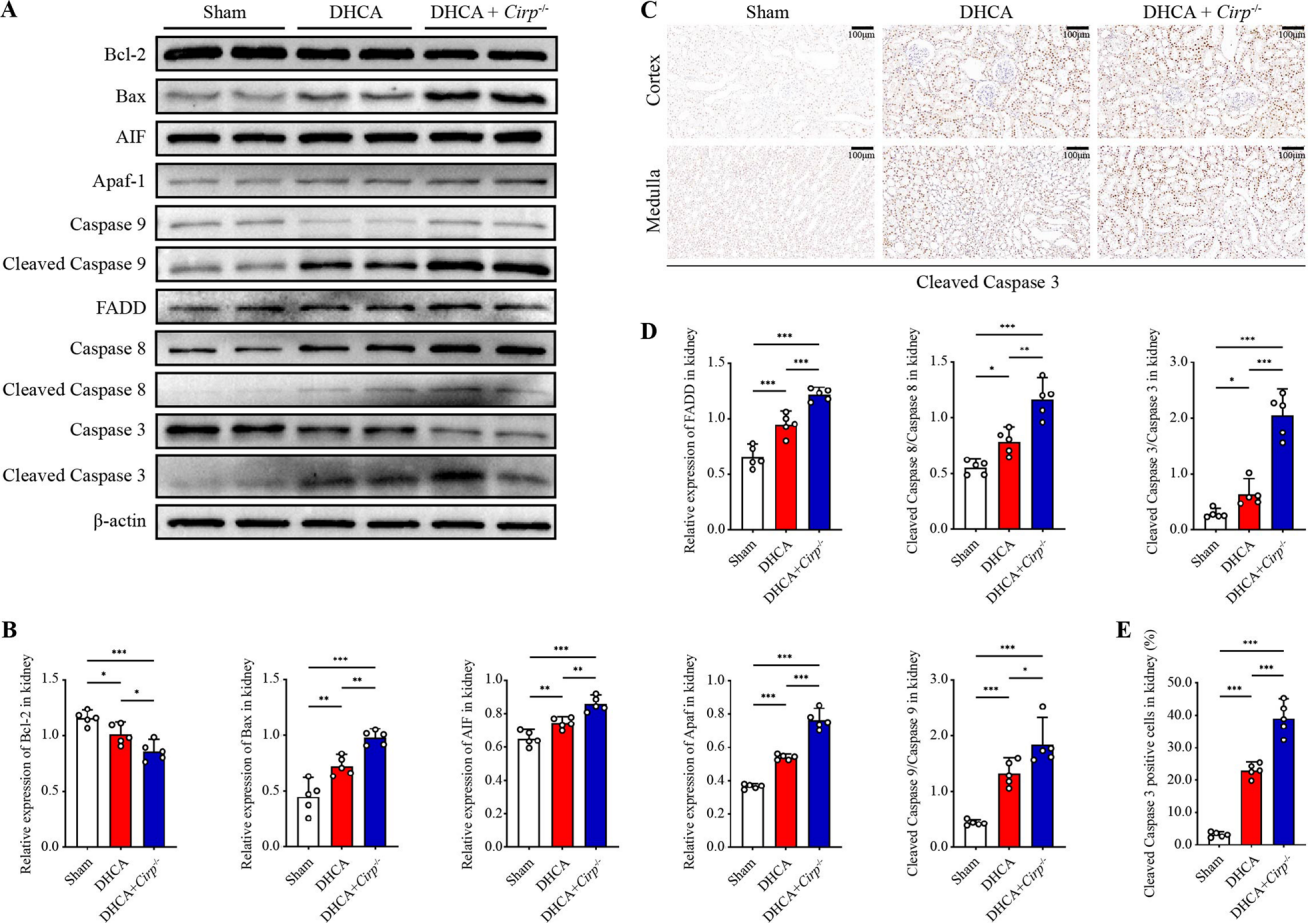
*Cirp* knockout exacerbated the mitochondrial apoptotic pathway and the death receptor apoptotic pathway after DHCA. **A** Western blot analyses of Bcl-2, Bax, AIF, Apaf-1, caspase 9 and cleaved caspase 9, FADD, caspase 8 and cleaved caspase 8 and caspase 3 and cleaved caspase 3 in kidneys from the sham, DHCA and DHCA + *Cirp*^*−/−*^ groups. **B** The ratio of key targets in the mitochondrial apoptotic pathway to β-actin by western blot analysis. **C** Representative immunohistochemistry images of cleaved caspase 3 in the renal cortex and medulla from the three groups (original magnification, × 200). **D** The ratio of key targets in death receptor apoptotic pathway to β-actin by western blot analysis. **E** The cleaved caspase 3-positive cells were quantified by the percentage in 5 randomly selected microscopic vision fields. Statistical significance was examined by one-way analysis of variance (ANOVA) followed by the Tukey test. *P < 0.05, **P < 0.01, ***P < 0.001

Furthermore, we examined the expression of the mitochondrial apoptotic pathway in renal tissue. Western blot analysis revealed that the expression levels of several key targets of the mitochondrial apoptotic pathway, including proapoptotic Bax, apoptosis-inducing factor (AIF), apoptotic protease activating factor-1 (Apaf-1) and activated cysteinyl aspartate specific proteinase (caspase) 9 (quantified as cleaved caspase 9/caspase 9), were significantly increased in the *Cirp*^*−/−*^ group, whereas antiapoptotic protein Bcl-2 was inhibited in the *Cirp*^*−/−*^ group compared to the DHCA group (P < 0.05, Fig. 5A, B).

Additionally, previous studies have reported that an enhanced inflammatory response and intensified DNA damage could activate the death receptor apoptotic pathway (Tummers et al. 2020; Norbury and Zhivotovsky 2004); therefore, we tested key targets in this pathway. The expression of Fas-associated death domain protein (FADD) and activated caspase 8 (quantified as cleaved caspase 8/caspase 8) was significantly increased in the *Cirp*^*−/−*^ group compared with the DHCA group (P < 0.05, Fig. 5A, D). Likewise, the activation of caspase 3, the joint endpoint of these two apoptotic pathways, was remarkably stronger in the *Cirp*^*−/−*^ group than in the DHCA group (P < 0.05, Fig. 5A, D). Moreover, immunohistochemistry of cleaved caspase 3 demonstrated a similar feature of changes among groups, which further confirmed these results (Fig. 5C, E).

In summary, based on the results of the DHCA model, we discovered that *Cirp* knockout may inhibit HIF-1α expression by stimulating PHD3 protein expression during DHCA. As a result, Trx-1 was restrained, thus reducing ROS removal and strengthening oxidative stress, which activated the TGF-β1/p38 MAPK inflammatory pathway. In a stepwise manner, mitochondrial damage and increased cytochrome c release were triggered, followed by mitochondrial apoptotic pathway activation. Meanwhile, damaged DNA accumulated as RPA2 activity was inhibited, which may synergistically activate death receptor apoptotic pathways, eventually rendering cell apoptosis and kidney injury.

### Apoptosis of HK-2 cells induced by hypothermic OGD was aggravated by *CIRP* knockdown but rescued by *PHD3* knockdown

To address whether the relationship between *CIRP* inhibition and TGF-β1/p38 MAPK activation was specifically mediated by the PHD3/HIF-1α axis and to elucidate whether the above mechanisms exist in human kidney cells, we established a hypothermic OGD model in HK-2 cells to simulate an in vivo hypothermic IR environment. Human siCIRP or siPHD3 was used to knock down the expression of each gene. Then, we detected the level of HIF-1α in untransfected HK-2 cells after exposure to hypoxia (1% O_2_) at 18 °C for 2, 4, 6, and 8 h, followed by reoxygenation for 30 min, to identify the optimal hypoxia protocol for OGD. Western blot analysis of whole-cell extracts demonstrated that HIF-1α was almost undetectable in normoxia and accumulated and peaked at 6 h after hypoxia followed by reoxygenation (Additional file 4: Figure S4A, B); this finding was corroborated by results in human microvascular endothelial cells (HMEC-1) of a peak HIF-1α protein level at 3–6 h of hypoxia (Chamboredon et al. 2011;). Therefore, all hypothermic OGD models were generated following the scheme of hypoxia for 6 h and reoxygenation for 30 min (Additional file 4: Figure S4C).

As shown in Additional file 4: Figure S4D-F, *CIRP* mRNA and CIRP protein were significantly induced after hypothermic OGD, whereas no significant difference in *PHD3* mRNA or PHD3 protein levels was noted among the control group, OGD group and OGD + siNC group (siNC group). The expression levels of *CIRP* and *PHD3* mRNA were successfully inactivated in the OGD + siCIRP group (siCIRP group) and the siPHD3 group (siPHD3 group), respectively, which was confirmed by the results at the protein level. Consistent with the results in rats, a significant increase in HIF-1α protein was observed in HK-2 cells after hypothermic OGD and was even more pronounced with *PHD3* defects (Additional file 4: Figure S4D-F). HIF-1α protein was significantly suppressed in the siCIRP group, as the CIRP/PHD3 axis was inhibited. Likewise, a similar expression pattern of the CIRP/PHD3/HIF-1α axis was validated by immunofluorescence (Additional file 4: Figure S4G-L). Nevertheless, there was no significant difference in the level of *HIF-1α* mRNA among the groups (Additional file 4: Figure S4D).

**Fig. 6:**
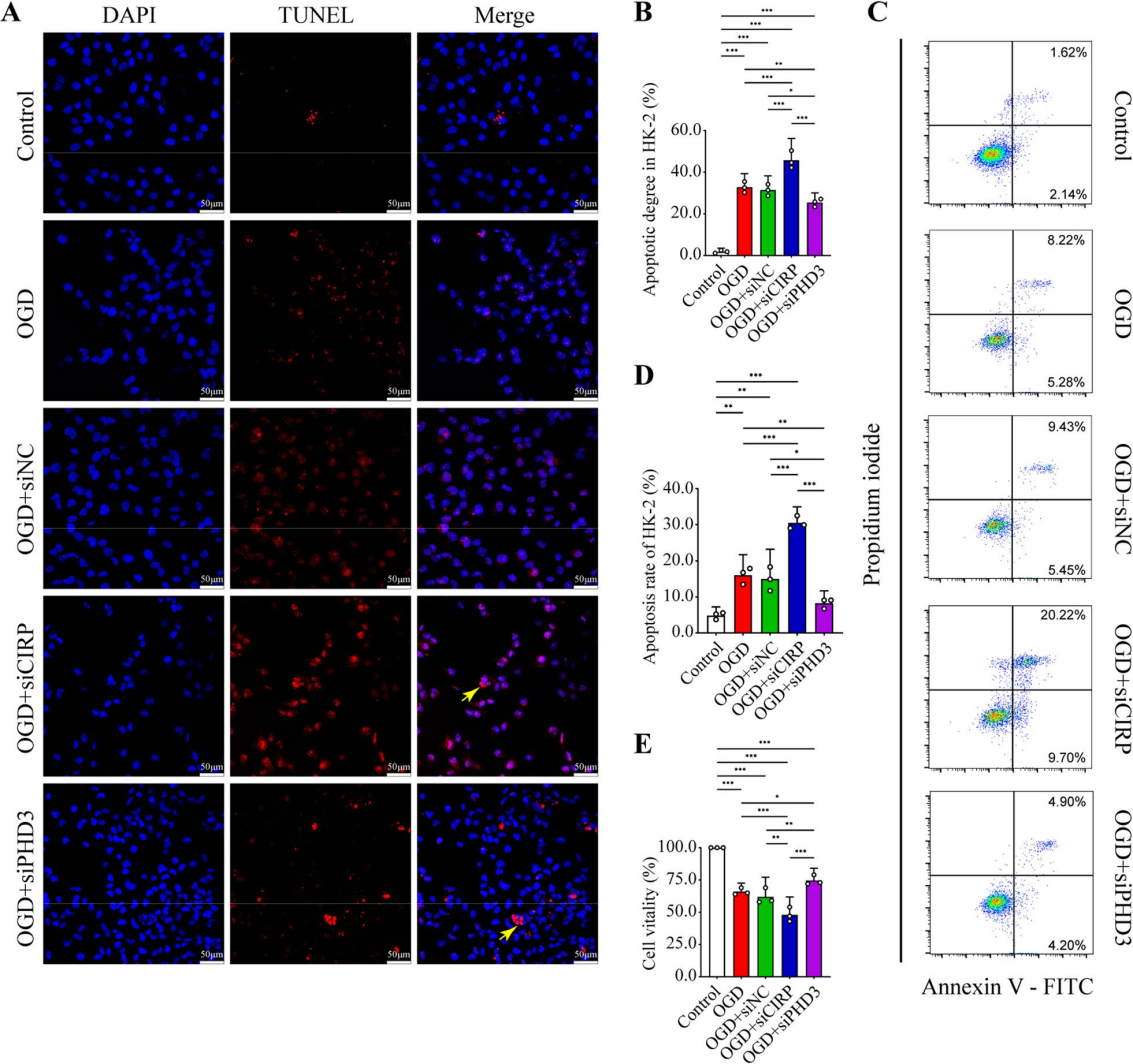
Apoptosis of HK-2 was aggravated by *CIRP* knockdown but attenuated by *PHD3* knockdown. **A** TUNEL staining of HK-2 in the control, OGD, OGD + siNC, OGD + siCIRP, OGD + siPHD3 groups (original magnification, × 400). Yellow arrows, apoptotic body. **B** The apoptotic degree was counted as the percentage of TUNEL signal in 5 randomly selected fields. **C, D** Apoptosis rate measurement by flow cytometry with Annexin V-FITC/propidium iodide (PI) double staining of HK-2 in five groups. **E** The cell vitality detection by cell counting kit (CCK)-8 of HK-2 in five groups. Statistical significance was examined by one-way analysis of variance (ANOVA) followed by the Tukey test. *P < 0.05, **P < 0.01, ***P < 0.001

To verify the role of the CIRP/PHD3/HIF-1α axis in OGD-induced apoptosis, we performed TUNEL and flow cytometry analyses. As shown in Fig. 6A, B, hypothermic OGD significantly prompted apoptosis, which was further aggravated by *CIRP* inactivation and its indirect suppression of HIF-1α. However, *PHD3* silencing and the alleviation of its inhibitory effect on HIF-α significantly blunted cell apoptosis after hypothermic OGD. Correspondingly, flow cytometry demonstrated that OGD markedly increased the apoptosis rate, and the increase was promoted by *CIRP* siRNA but ameliorated by *PHD3* ablation (Fig. 6C, D). Meanwhile, the apoptosis rate was comparable between the OGD group and the siNC group. In addition, cell vitality was significantly impaired in the siCIRP group compared with the OGD group and siNC group. Nevertheless, the OGD-induced decrease in cell vitality was significantly reversed by HIF-1α accumulation in the siPHD3 group (Fig. 6E).

These results illuminated that CIRP/PHD3 axis may exert protective effect through preserving HIF-1α against OGD-induced apoptosis of HK-2 cells.

### *CIRP* knockdown accumulated ROS and activated the inflammatory pathway in HK-2 cells via the PHD3/HIF-1α axis

To examine how CIRP/PHD3/HIF-1α mediated the apoptosis of HK-2 cells, we evaluated the levels of ROS and inflammatory cytokines. According to fluorescent DCFH-DA detection, ROS increased in the OGD and siNC groups, in comparison to the control group (Additional file 5: Figure S5A&B). Concurrently, this increase was strengthened by HIF-1α degradation in the siCIRP group but was attenuated by HIF-1α accumulation in the siPHD3 group. ELISA revealed a parallel trend of IL-1β, IL-6 and TNF-α levels in the cell lysate and culture medium among the groups (Additional file 5: Figure S5C). Because ROS is a pivotal activator of the p38 MAPK pathway in oxidative stress (Foerster et al. 2022), we next measured the expression of the TGF-β1 and pp38 MAPK/p38 MAPK in HK-2 cells. The results showed that TGF-β1/p38 MAPK pathway was significantly activated in the siCIRP group but inhibited in the siPHD3 group compared with the OGD group (Additional file 5: Figure S5D, E). In addition, we analyzed the expression of Trx-1 and found that after OGD, Trx-1 was significantly attenuated in the siCIRP group and was increased in the siPHD3 group (Additional file 5: Figure S5D, E). The alteration of ROS clearance caused by the variation in Trx-1 expression may further explain the above results. Our next question was whether the activation of the above inflammatory pathway would cause mitochondrial damage. Therefore, we next detected MMP in HK-2 cells, the decrease of which may indicate mitochondrial damage. Likewise, the results showed that OGD significantly reduced MMP and ATP production, which was exacerbated by *CIRP* knockdown, while *PHD3* knockdown alleviated the MMP and ATP content decline caused by OGD (Additional file 5: Figure S5F&G).

Collectively, the mechanism by which the CIRP/PHD3/HIF-1α axis activates the inflammatory pathway and damages mitochondria by attenuating ROS quenching in rat kidneys under hypothermic and hypoxic conditions may analogously exist in HK-2 cells.

### The CIRP/PHD3/HIF-1α axis aggravated apoptosis of HK-2 through the mitochondrial pathway and death receptor pathway

Subsequently, we tested the expression of extramitochondrial cytochrome c released by ruptured mitochondria in vitro, as well as the degree of activation of apoptotic pathway. As Additional file 6: Figure S6A, B, D showed, the level of extramitochondrial cytochrome c increased significantly in the siCIRP group as did the levels of its downstream molecules Apaf-1, cleaved caspase 9 and cleaved caspase 3 in the mitochondrial apoptotic pathway, compared with the OGD group. However, downregulation of *PHD3* significantly suppressed the mitochondrial apoptotic pathway after OGD. Moreover, the results shown in Additional file 6: Figure S6A, C validated our hypothesis that *CIRP* knockdown in the OGD model restrains the expression of RPA2, which is responsible for repairing damaged DNA. *CIRP* knockdown may also elevate Bax expression while lowering Bcl-2 expression, thus promoting an increase in mitochondrial permeability and the release of AIF, which eventually contributed to cell apoptosis. Nevertheless, *PHD3* knockdown in the OGD model significantly reversed these trends (Additional file 6: Figure S6A, C). Furthermore, there were significant increases in the expression of the key targets of the death receptor pathway, FADD and cleaved caspase 8 in the siCIRP group compared with the OGD group (Additional file 6: Figure S6A, D). In contrast, the death receptor pathway was markedly impeded in the siPHD3 group after hypothermic OGD compared with that in untransfected cells.

In summary, these results indicated that CIRP could relieve apoptosis of renal cells by indirectly increasing the expression of HIF-1α under conditions of hypothermia and hypoxia, which was consistent with the in vivo study.

### Enarodustat alleviated the apoptosis aggravated by CIRP expurgation both in vitro and in vivo

**Fig. 7:**
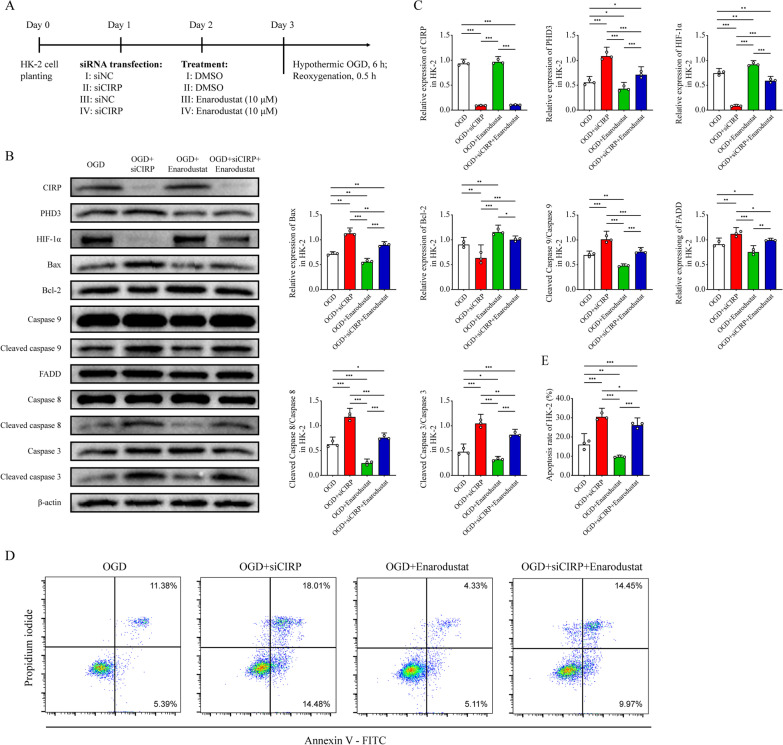
Enarodustat reversed HK-2 apoptosis aggravated by *CIRP* knockdown and attenuated HK-2 apoptosis in hypothermic OGD. **A** Experimental protocol of siRNA transfection and enarodustat or vehicle pretreatment of HK-2 cells. **B, C** Western blot analyses of CIRP, PHD3, HIF-1α, Bax, Bcl-2, caspase 9 and cleaved caspase 9, FADD, caspase 8 and cleaved caspase 8, caspase 3 and cleaved caspase 3 and β-actin of HK-2 in the OGD, OGD + siCIRP, OGD + enarodustat and OGD + siCIRP + Enarodustat groups. **D, E** Apoptosis rate measurement by flow cytometry with Annexin V-FITC/propidium iodide (PI) double staining of HK-2 in four groups. Statistical significance was examined by one-way analysis of variance (ANOVA) followed by the Tukey test. *P < 0.05, **P < 0.01, ***P < 0.001

Next, we explored the effect of HIF-1α stabilizer enarodustat (JTZ-951, Selleck, Houston, USA) on HK-2 cells in hypothermic OGD (Fig. 7A). Enarodustat pretreatment (10 μM) remarkably upregulated HIF-1α compared with OGD group (Fig. 7B, C). Besides, enarodustat significantly reversed the apoptosis degree aggravated by *CIRP* knockdown, manifested as increased activation of Bax, caspase 9, FADD, caspase 8 and caspase 3 and inhibited expression of Bcl-2. Furthermore, enarodustat pretreatment exerted an anti-apoptotic effect on HK-2 undergoing hypothermic OGD compared with vehicle (DMSO) group. The results of flow cytometry revealed the similar trend between groups (Fig. 7D, E).

**Fig. 8:**
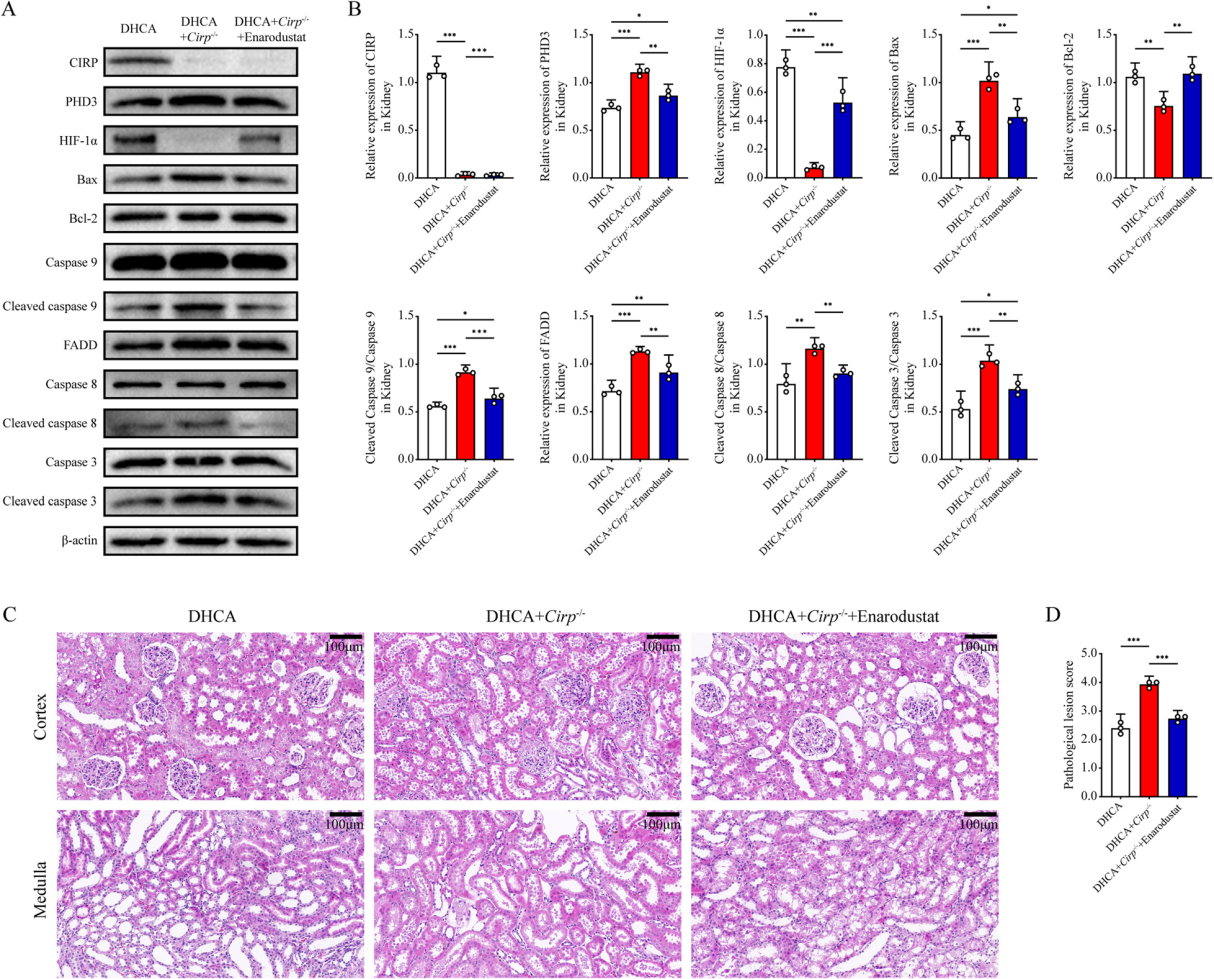
Enarodustat alleviated renal injury aggravated by *Cirp* knockout in rats undergoing DHCA. **A, B** Western blot analyses of CIRP, PHD3, HIF-1α, Bax, Bcl-2, caspase 9 and cleaved caspase 9, FADD, caspase 8 and cleaved caspase 8, caspase 3 and cleaved caspase 3 and β-actin of rats in the DHCA, DHCA + *Cirp*^*−/−*^ and DHCA + *Cirp*^*−/−*^ + Enarodustat groups. **C** Representative H&E staining images of the renal cortex and medulla in the DHCA, DHCA + *Cirp*^*−/−*^ and DHCA + *Cirp*^*−/−*^ + Enarodustat groups (original magnification, × 200). **D** Pathological lesion score of renal samples from three groups. Statistical significance was examined by one-way analysis of variance (ANOVA) followed by the Tukey test. *P < 0.05, **P < 0.01, ***P < 0.001

On this basis, we established rat DHCA models in order to explore whether enarodustat could exert similar effects in vivo (n = 3 in each group). Enarodustat or vehicle was administered by oral gavage at the daily dose of 3 mg/kg from 5 days before DHCA operation to the day of the operation. Compared with wild-type rats, *Cirp* knockout significantly exacerbated apoptosis degree and renal injury of rats, which was counteracted by enarodustat (Fig. 8, Additional file 7: Figure S7).

Collectively, HIF-1α stabilizer enarodustat could mitigate renal apoptosis aggravated by CIRP ablation, which confirmed the above results that CIRP mediated renal hypothermic IR injury (IRI) via the PHD3/HIF-1α axis.

## Discussion

**Fig. 9:**
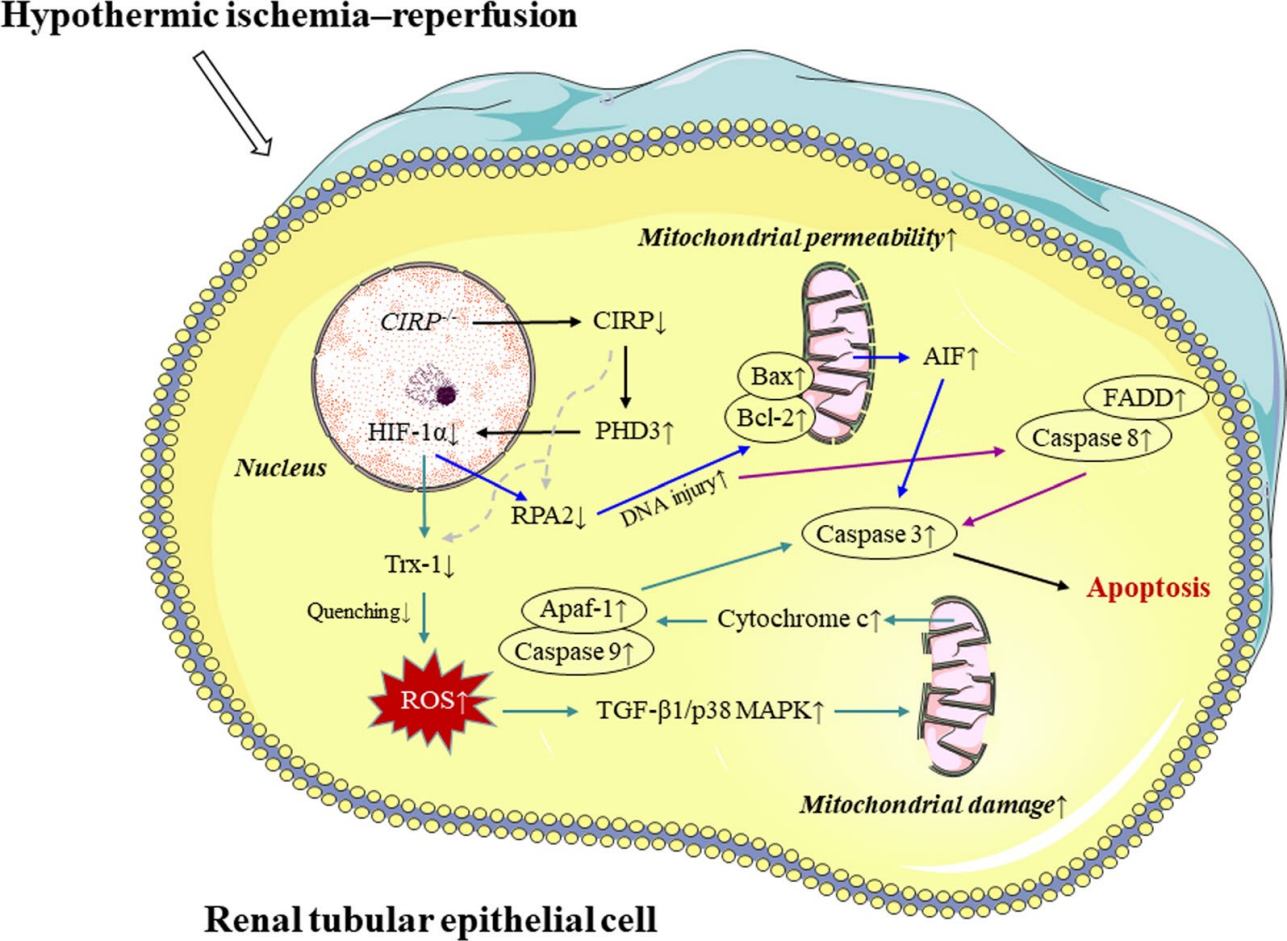
Putative mechanisms of *CIRP*^*−/−*^ on aggravating apoptosis in renal tubular epithelial cell during hypothermic ischemia**–**reperfusion injury. The diagrams of cytomembrane, nucleus and complete mitochondria was provided by Servier Medical Art (https://smart.servier.com) under CC BY 3.0 license

In the present study, we used a genetic approach combined with transcriptome sequencing to identify an underlying mechanism linking CIRP to renal protection during hypothermic IR (Fig. 9). To the best of our knowledge, this study is the first to provide in vivo and in vitro mechanism evidence that CIRP regulates the level of oxidative stress and the expression of inflammatory pathways during hypothermic IR via the PHD3/HIF-1α axis and inhibits endogenous and exogenous apoptotic pathways, thereby exerting the renal anti-apoptotic effect. Our results indicated that CIRP is a latent therapeutic target for alleviating AKI after DHCA through a PHD3/HIF-1α-dependent mechanism.

It is worth noting that, different from the common rat renal IR model induced by short-term (< 1 h) clamping of renal pedicle followed by removal of forceps in previous studies, the pathophysiological process of renal injury during DHCA operation involves multi-mechanisms not only IR, but also hypothermia, hypoxia, hemodynamic disorder and blood destruction. In rat study, we observed significant manifestations of renal injury and apoptosis after one hour of DHCA followed by rewarming and reperfusion. In accessible studies focusing on rat acute brain or cardiac injury after DHCA operation, similar or even higher apoptosis rate was found (Liu et al. 2019, 2020). These results highlight the importance of exploring the intrinsic mechanism to prevent IRI after DHCA in time.

CIRP, a member of the cold shock protein family, can be significantly induced in response to cellular stress (Zhu et al. 2016; Liao et al. 2017). In this study, CIRP protein increased rapidly in renal tissue and tubular epithelial cells after hypothermic IR to play an anti-cellular damage role. This finding indicated that CIRP is an acute phase protein in renal cells under hypothermic and hypoxic stress. We analyzed the difference in gene expression between wild-type and *Cirp*^*−/−*^ rats after DHCA by RNA-sequencing. After screening the DEGs by qPCR experiments, we originally discovered that *Cirp* can act as an inhibitory upstream regulator of the *Phd3* gene. Cell experiments had corresponding results: PHD3 content was significantly increased in HK-2 cells transfected with siCIRP after simulation with hypothermic IR compared with untransfected cells.

PHD3 is a specific regulator of HIF-1α (Wenger and Hoogewijs 2010) and is expressed in kidneys (Haase 2013). Interestingly, even though HIF-1α is the core molecule of oxygen regulation, it cannot directly sense alterations of oxygen tension. Instead, its expression is controlled by the oxygen sensor PHD3. In a normoxic environment, the proline residues of HIF-1α subunits are degraded by oxygen-dependent PHD3 hydroxylation (Wenger and Hoogewijs 2010). However, under hypoxia, the activity of PHD3 is inhibited, and the HIF-1α subunit and HIF-1β form dimers and are stably expressed to regulate genes related to hypoxia adaptation (Lee et al. 2019; Bruzzese et al. 2021; Fu et al. 2020; Ito et al. 2020). Both in vivo and in vitro, no marked change in *HIF-1α* mRNA level was observed when *CIRP* or *PHD3* was downregulated; however, the level of HIF-1α protein was significantly inhibited by PHD3. This confirmed that the inhibition of HIF-1α by PHD3 involved posttranslational regulation.

Previous studies have shown that HIF-1α can confer a protective effect against renal IRI (Fu et al. 2020; Rosenberger et al. 2002;). In particular, specifical inhibition of PHD alleviated IR-induced AKI (Bernhardt et al. 2006; Rosenberger et al. 2008). In this study, we offered supportive evidence that the CIRP/PHD3/HIF-1α axis is a regulator of renal cell apoptosis, which can attenuate posthypothermic IR renal injury by inhibiting oxidative stress and the inflammatory pathway. Trx-1 is a specific scavenger of ROS (Yang et al. 2006), which is a recognized marker of oxidative stress levels. We found that when HIF-1α was indirectly inhibited by *CIRP* ablation, the expression of Trx-1 decreased significantly, resulting in ROS accumulation and activation of the TGF-β1/p38 MAPK inflammatory pathway, thereby contributing to the rupture of the mitochondrial membrane and the release of cytochrome c. The outflow of cytochrome c and an increase in the proapoptotic signal (Bax/Bcl-2) led to mitochondrial vacuolation and swelling with a decrease in MMP (Plotnikov et al. 2007), thus impairing the function and structural integrity of renal tubular epithelial cells (Fu et al. 2020; Schrier et al. 2004). Correspondingly, we observed obvious swelling and ruptured mitochondria in renal tubular epithelial cells when HIF-1α was inhibited, as a previous study reported (Fu et al. 2020). In damaged mitochondria, the interruption of electron transport in the respiratory chain prompts ROS generation (Zorov et al. 2014), and, as a result of this, in our study, we observed initiation of a positive feedback loop of ROS accumulation and mitochondrial damage. Additionally, the decrease in RPA2 caused by inhibition of HIF-1α weakened the repair of damaged DNA, as a previous study reported (Givalos et al. 2007), which contributed to the change in mitochondrial permeability and the release of AIF. Cytochrome c and AIF jointly activate the mitochondrial apoptosis pathway and cause renal cell apoptosis. Meanwhile, apoptosis of renal cells caused by a decrease in HIF-1α has also been found to be involved in the activation of the death receptor apoptotic pathway either in vivo or in vitro. Notably, when PHD3 was silenced in HK-2 cells, the increase in HIF-1α remarkably reversed the above effects. Therefore, these results revealed a plausible route for CIRP in mitigating renal hypothermic IRI, that is, to inhibit endogenous (mitochondrial) and exogenous (death receptor) apoptotic pathways through the PHD3/HIF-1α axis.

This study adds evidence that implicates HIF-1α stabilizer in renal injury in various clinical conditions. Enarodustat, a HIF-1α stabilizer, is already experimentally used for renal anemia in several ongoing clinical trials (Semenza 2014; Sugahara et al. 2017; Hasegawa et al. 2018). In vitro and in vivo data supported HIF-1α stabilizer as prophylactic treatment for AKI clinically in major surgery (Ito et al. 2020). In a rat model of renal IRI, enarodustat conferred renal protection by increasing glycogen storage in kidneys (Ito et al. 2020). Moreover, enarodustat has been reported to suppress oxidative stress in renal tissue and to improve renal function in diabetic rats (Hasegawa et al. 2020). In this study, our results provide experimental evidence for enarodustat against AKI after hypothermic cardiovascular surgery, which may expand the potential application value of HIF-1α stabilizers.

Certain limitations of this study should be mentioned. First, we did not perform in vivo experiments of *PHD3* gene expression interference, which would provide definitive evidence for the role of the CIRP/PHD3/HIF-1α axis in renal hypothermic IRI. Second, CIRP has been reported to protect cells from injury by directly promoting the expression of RPA2 and Trx-1 under UV stimulation (Yang and Carrier 2001). However, under the conditions of hypothermia and hypoxia in this study, the expression levels of RPA2 and Trx-1 in HK-2 cells were significantly increased when *PHD3* was silenced. This suggested that PHD3 was the main instigator of the changes in RPA2 and Trx-1 levels. Third, our study could not describe the effect of extracellular CIRP, which has been proven in some studies to act as a damage-associated molecular pattern (DAMP) in prompting inflammation and inducing apoptosis (Liao et al. 2017; Aziz et al. 2019). Fourth, the wild-type and *Cirp*^*−/−*^ rats with a SD background used in this study belong to outbred strains. These strains of rats may have varying susceptibility to ischemic kidney injury, and cross-breeding would alleviate the interference caused by individual differences. Fifth, this study focused on the occurrence and mechanism of renal apoptosis. We cannot determine whether renal apoptosis is accompanied by other types of cell death, such as necrosis, pyroptosis, ferroptosis and autophagy in this study. Sixth, according to the detection of western blot, ELISA, qRT-PCR and RNA-sequencing of apoptosis pathway in rat kidney, the kidney was identified as a whole that experienced hypothermic IR in this study, and the degree of apoptosis between cortex and medulla was not distinguished. Last, as the first preliminary exploration of the underlying mechanisms of AKI after DHCA using both in vivo and in vitro models, the sample size in animal experiments was based on previous studies and our past experience using this animal model (Liu et al. 2020; Li et al. 2019).

## Conclusions

In summary, our study provides experimental evidence that CIRP exerts a protective effect against AKI through PHD3/HIF-1α mediated apoptosis in DHCA. HIF-1α stabilizers may serve as a potential therapeutic measure to improve outcomes in renal hypothermic IRI.

## Supplementary Information

**Additional file 1: Figure S1.** Schematic diagram of rat deep hypothermic circulatory arrest model. O_2_, oxygen. CO_2_, carbon dioxide.

**Additional file 2: Figure S2.** Photograph of rat deep hypothermic circulatory arrest model in action.

**Additional file 3: Figure S3.** RNA-sequencing analysis of renal tissues from wild-type and *Cirp*^−/−^ rats after DHCA. **A** Volcano plot of differentially expressed genes (DEGs). Grey dots, non-DEGs (21523 genes). Red dots, upregulated DEGs (190 genes). Blue dots, downregulated DEGs (113 genes). **B**–**D** Gene Ontology (GO) analysis including biological process (BP), cellular componentand (CC) molecular functionof the identified DEGs. **E** The mostly enriched pathways of DEGs clustered by Kyoto Encyclopedia of Genes and Genomes (KEGG) analysis.

**Additional file 4: Figure S4.** *CIRP* knockdown activated PHD3/HIF-1α axis in HK-2 cells after hypothermic OGD. **A**, **B** Representative image of HIF-1α and ratio of HIF-1α/β-actin after 0, 2, 4, 6, 8 h of hypothermic oxygen and glucose deprivation (OGD) followed by 0.5 h of reoxygenation by western blot. **C** The pattern of hypothermic ischemia–reperfusion in HK-2 cells. **D** Levels of *CIRP*/β-actin, *PHD3*/β-actin and *HIF-1α*/β-actin mRNA in HK-2 from the control, OGD, OGD + siNC, OGD + siCIRP, OGD + siPHD3 groups (triplicate per group). **E** Western blot analyses of CIRP, PHD3 and HIF-1α in the five groups. **F** The ratio of CIRP, PHD3 or HIF-1α to β-actin by western blot analysis. **G**, **H**, **I** Representative immunofluorescence images of CIRP, PHD3 and HIF-1α in HK-2 from the five groups (original magnification, ×400). **J**, **K**, **L** The CIRP, PHD3 or HIF-1α-positive cells were quantified by the percentage in 5 randomly selected microscopic vision fields. Statistical significance was examined by one-way analysis of variance (ANOVA) followed by the Tukey test. *P < 0.05, **P  < 0.01, ***P < 0.001.

**Additional file 5: Figure S5.** *CIRP* knockdown accumulated ROS and activated the inflammatory pathway in HK-2 via PHD3/HIF-1α axis. **A** ROS detection by DCFH-DA of HK-2 in the control, OGD, OGD + siNC, OGD + siCIRP, OGD + siPHD3 groups (original magnification, ×200). **B** Fluorescence intensity of ROS in HK-2 from the five groups. **C** ELISA analyses of IL-1β, IL-6 and TNF-α in cell lysate and culture medium among groups. **D** Western blot analyses of Trx-1, TGF-β1, p38 MAPK and phospho-p38 (pp38) MAPK in five groups. **E** The ratio of Trx-1 or TGF-β1 to β-actin and pp38 MAPK/p38 MAPK by western blot analysis. **F** Mitochondrial membrane potential (MMP) measurement of HK-2 in the five groups. G ATP contents of HK-2 in the five groups. ROS, reactive oxygen species. Statistical significance was examined by one-way analysis of variance (ANOVA) followed by the Tukey test. *P < 0.05, **P  <  0.01, ***P < 0.001.

**Additional file 6: Figure S6.** CIRP/PHD3/HIF-1α axis aggravated apoptosis of HK-2 through the mitochondrial pathway and death receptor pathway. **A** Western blot analyses of extramitochondrial cytochrome c, total Apaf-1, caspase 9 and cleaved caspase 9, RPA2, Bax, Bcl-2, AIF, FADD, caspase 8 and cleaved caspase 8, caspase 3 and cleaved caspase 3 of HK-2 in the control, OGD, OGD + siNC, OGD + siCIRP, OGD + siPHD3 groups. **B** The ratio of extramitochondrial cytochrome c, Apaf-1 and cleaved caspase 9/caspase 9 by western blot analysis. **C** The ratio of RPA2, Bax, Bcl-2, AIF to β-actin by western blot analysis. **D** The ratio of FADD/β-actin and cleaved caspase 8/caspase8, cleaved caspase 3/caspase 3 by western blot analysis. Statistical significance was examined by one-way analysis of variance (ANOVA) followed by the Tukey test. *P < 0.05, **P < 0.01, ***P < 0.001.

**Additional file 7: Figure S7.** Biochemical index measurement of rat renal function. SCr, serum creatinine. BUN, blood urea nitrogen. DHCA, deep hypothermic circulatory arrest.

**Additional file 8: Table S1.** Sequences of primers used for qRT–PCR. **Table S2.** Primary and secondary antibodies. **Table S3.** Perioperative physiological index monitoring and blood-gas analysis.

## Data Availability

Not applicable.

## Abbreviations

DHCA: Deep hypothermic circulatory arrest
AKI: Acute kidney injury
CIRP: Cold inducible RNA-binding protein
IRI: Ischemia–reperfusion injury
CPB: Cardiopulmonary bypass
DEG: Differentially expressed gene
GO: Gene ontology
KEGG: Kyoto Encyclopedia of Genes and Genomes
SCr: Serum creatinine
BUN: Blood urea nitrogen
PHD3: Prolyl hydroxylase 3
TUNEL: TdT-mediated dUTP nick end labeling
ROS: Reactive oxygen species
HIF-1α: Hypoxia-inducible factor 1α
TEM: Transmission electron microscopy
