# Supplementary figures and images for: CIRP attenuates acute kidney injury after hypothermic cardiovascular surgery by inhibiting PHD3/HIF-1α-mediated ROS-TGF-β1/p38 MAPK activation and mitochondrial apoptotic pathways
Source: Mol Med. 2023 May 1;29:61. doi: 10.1186/s10020-023-00655-0 (PMC10152741; doi:10.1186/s10020-023-00655-0)

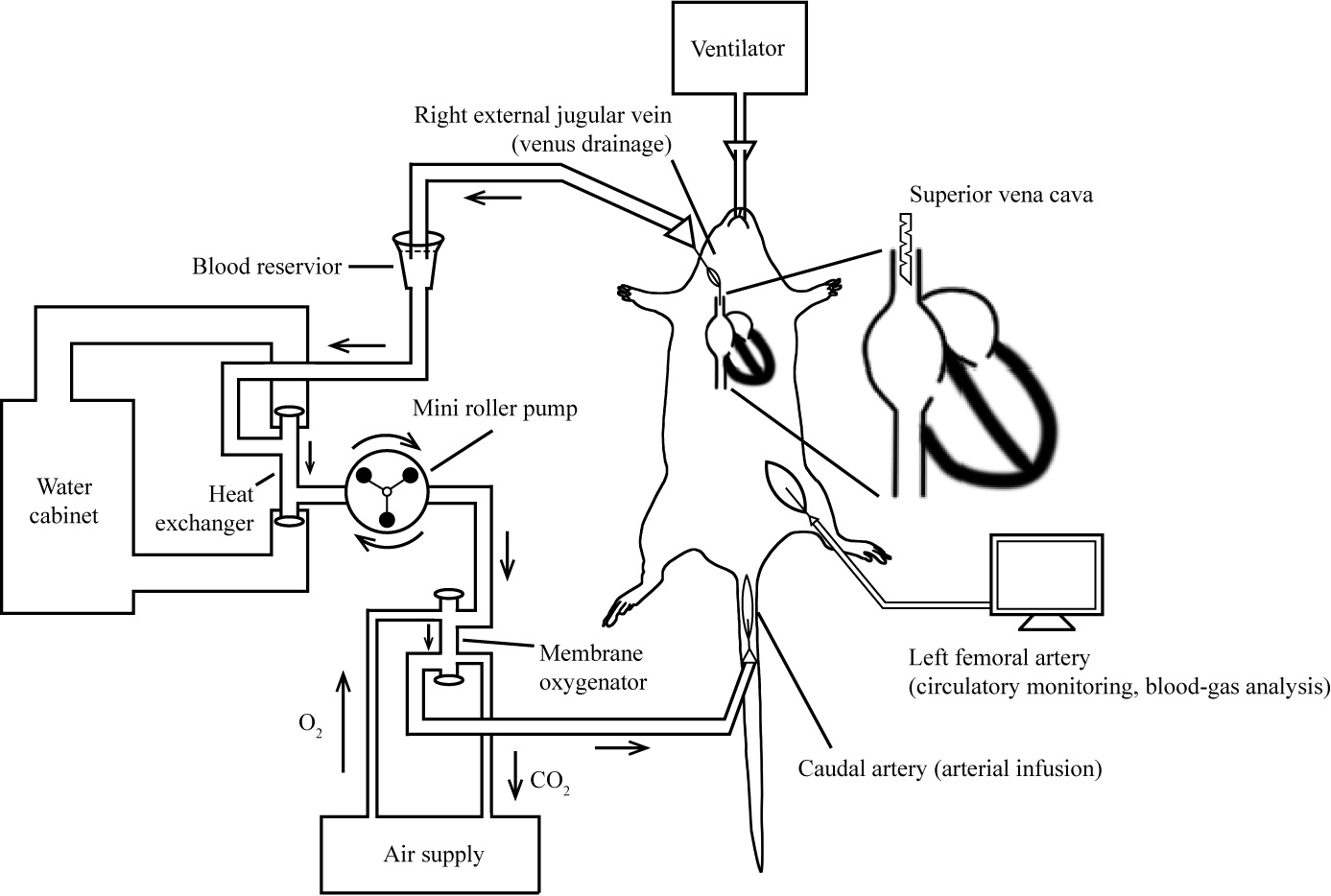

Supplement: Supplementary file 1 — Additional file 1: Figure S1. Schematic diagram of rat deep hypothermic circulatory arrest model. O2, oxygen. CO2, carbon dioxide. [file 10020_2023_655_MOESM1_ESM.jpg]

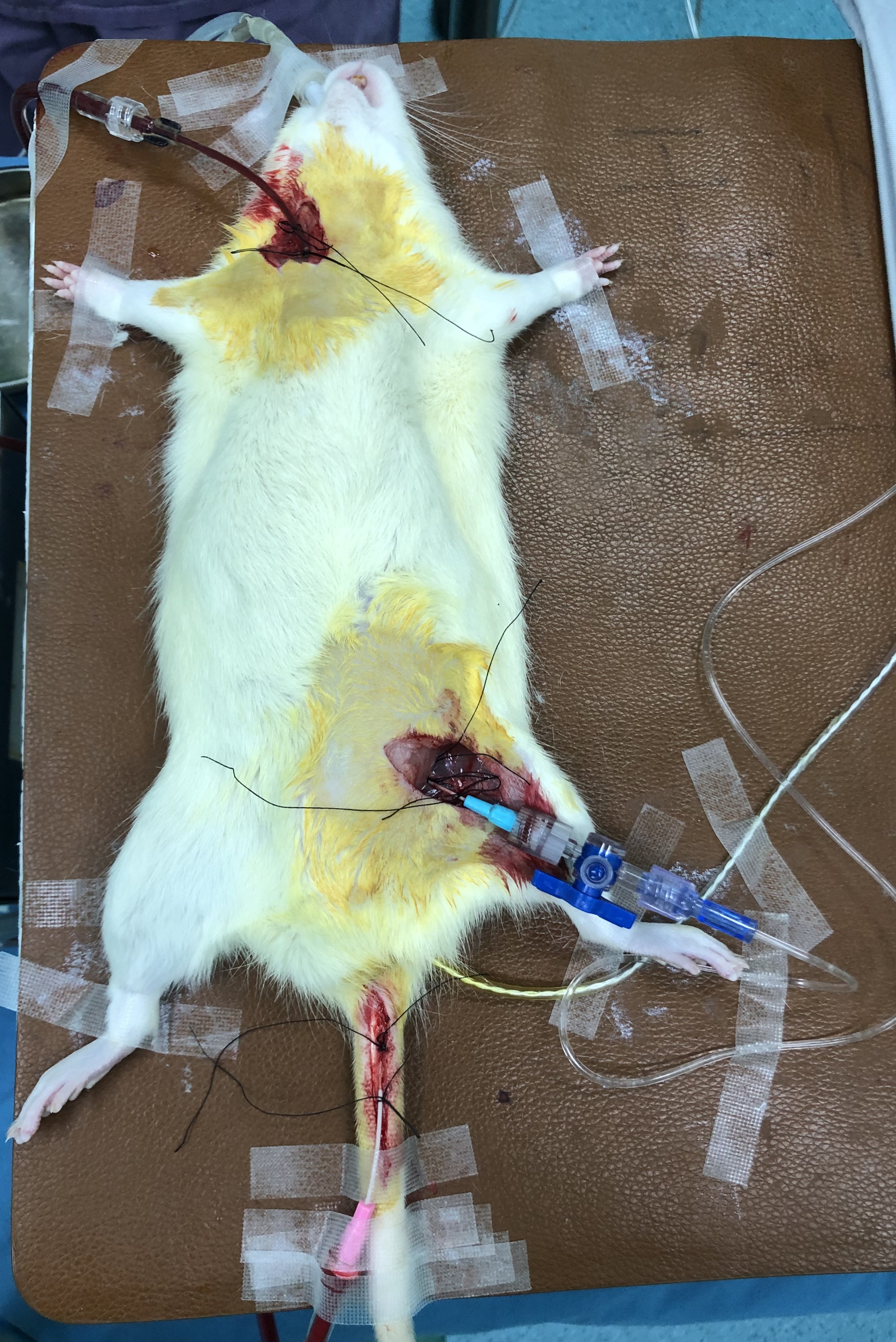

Supplement: Supplementary file 2 — Additional file 2: Figure S2. Photograph of rat deep hypothermic circulatory arrest model in action. [file 10020_2023_655_MOESM2_ESM.jpg]

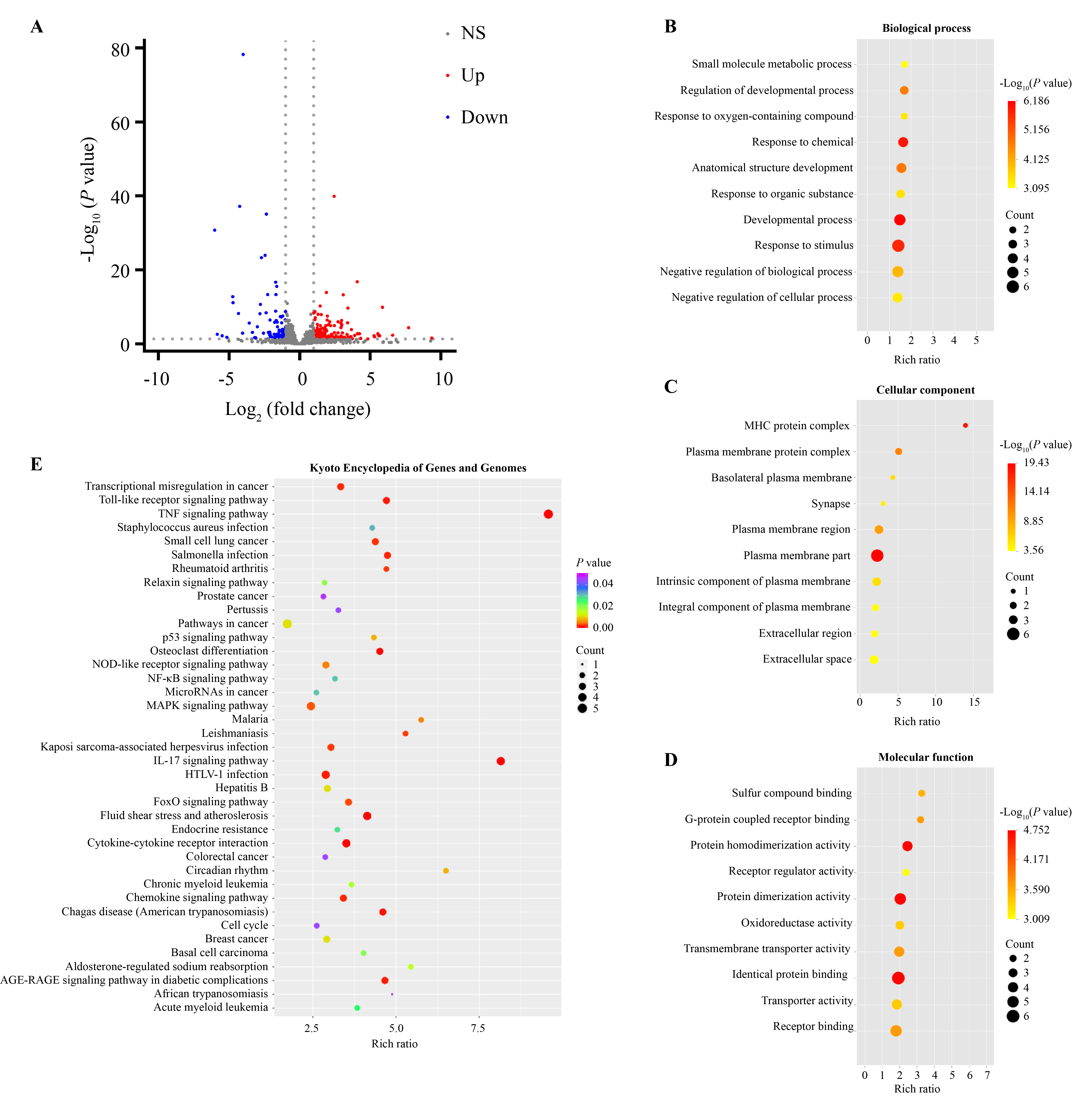

Supplement: Supplementary file 3 — Additional file 3: Figure S3. RNA-sequencing analysis of renal tissues from wild-type and Cirp−/− rats after DHCA. A Volcano plot of differentially expressed genes (DEGs). Grey dots, non-DEGs (21523 genes). Red dots, upregulated DEGs (190 genes). Blue dots, downregulated DEGs (113 genes). B–D Gene Ontology (GO) analysis including biological process (BP), cellular componentand (CC) molecular functionof the identified DEGs. E The mostly enriched pathways of DEGs clustered by Kyoto Encyclopedia of Genes and Genomes (KEGG) analysis. [file 10020_2023_655_MOESM3_ESM.jpg]

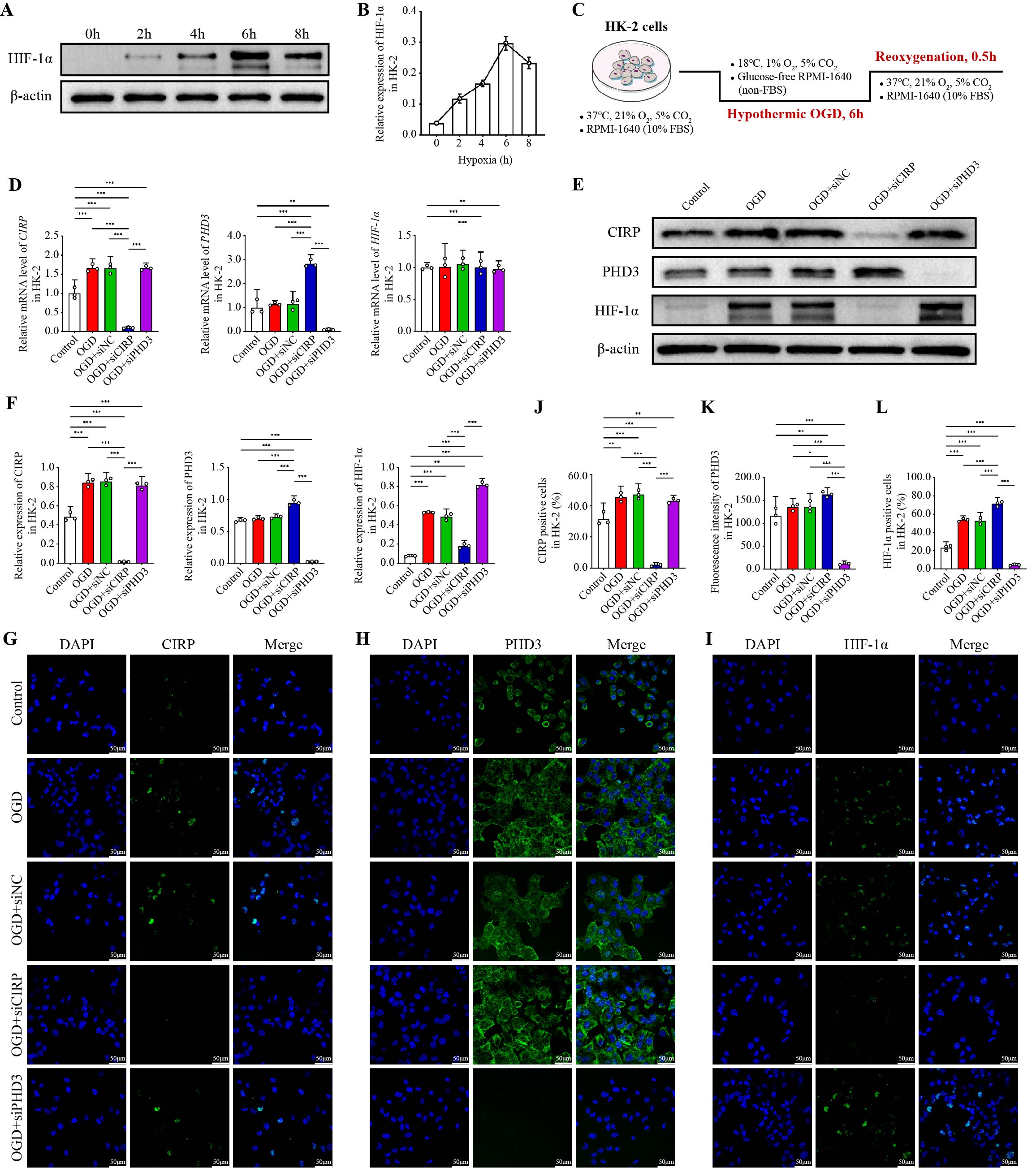

Supplement: Supplementary file 4 — Additional file 4: Figure S4. CIRP knockdown activated PHD3/HIF-1α axis in HK-2 cells after hypothermic OGD. A, B Representative image of HIF-1α and ratio of HIF-1α/β-actin after 0, 2, 4, 6, 8 h of hypothermic oxygen and glucose deprivation (OGD) followed by 0.5 h of reoxygenation by western blot. C The pattern of hypothermic ischemia–reperfusion in HK-2 cells. D Levels of CIRP/β-actin, PHD3/β-actin and HIF-1α/β-actin mRNA in HK-2 from the control, OGD, OGD + siNC, OGD + siCIRP, OGD + siPHD3 groups (triplicate per group). E Western blot analyses of CIRP, PHD3 and HIF-1α in the five groups. F The ratio of CIRP, PHD3 or HIF-1α to β-actin by western blot analysis. G, H, I Representative immunofluorescence images of CIRP, PHD3 and HIF-1α in HK-2 from the five groups (original magnification, ×400). J, K, L The CIRP, PHD3 or HIF-1α-positive cells were quantified by the percentage in 5 randomly selected microscopic vision fields. Statistical significance was examined by one-way analysis of variance (ANOVA) followed by the Tukey test. *P < 0.05, **P < 0.01, ***P < 0.001. [file 10020_2023_655_MOESM4_ESM.jpg]

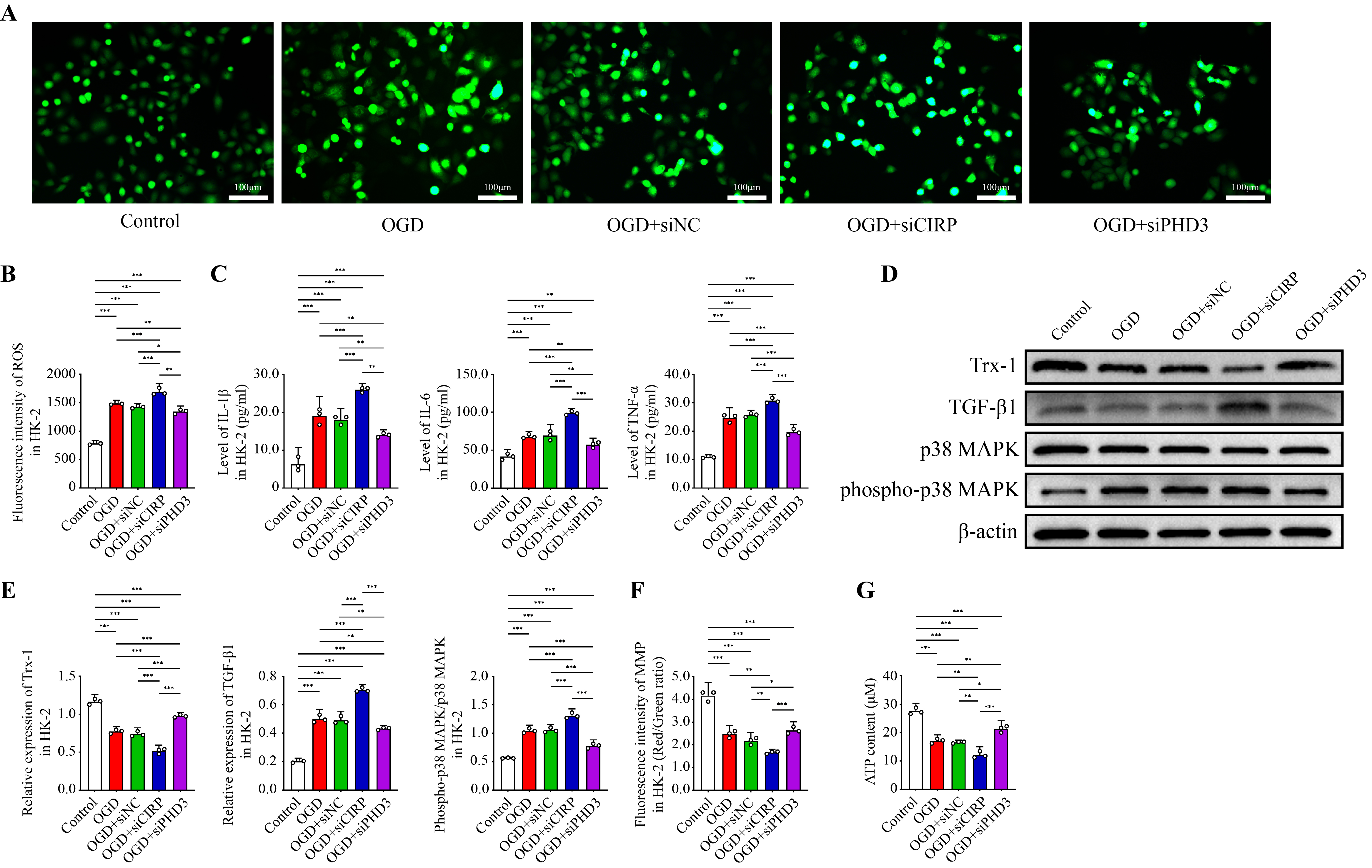

Supplement: Supplementary file 5 — Additional file 5: Figure S5. CIRP knockdown accumulated ROS and activated the inflammatory pathway in HK-2 via PHD3/HIF-1α axis. A ROS detection by DCFH-DA of HK-2 in the control, OGD, OGD + siNC, OGD + siCIRP, OGD + siPHD3 groups (original magnification, ×200). B Fluorescence intensity of ROS in HK-2 from the five groups. C ELISA analyses of IL-1β, IL-6 and TNF-α in cell lysate and culture medium among groups. D Western blot analyses of Trx-1, TGF-β1, p38 MAPK and phospho-p38 (pp38) MAPK in five groups. E The ratio of Trx-1 or TGF-β1 to β-actin and pp38 MAPK/p38 MAPK by western blot analysis. F Mitochondrial membrane potential (MMP) measurement of HK-2 in the five groups. G ATP contents of HK-2 in the five groups. ROS, reactive oxygen species. Statistical significance was examined by one-way analysis of variance (ANOVA) followed by the Tukey test. *P < 0.05, **P < 0.01, ***P < 0.001. [file 10020_2023_655_MOESM5_ESM.jpg]

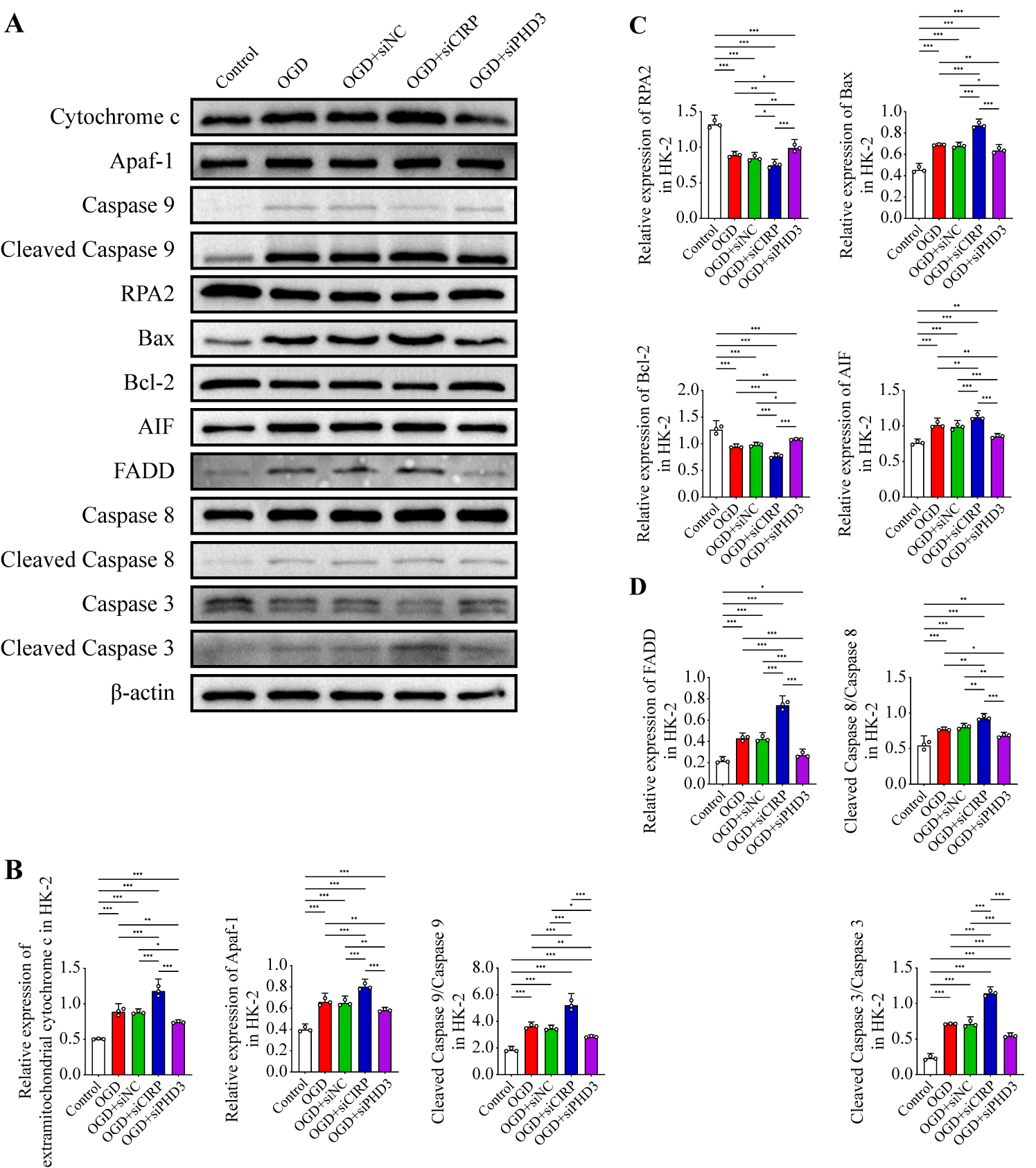

Supplement: Supplementary file 6 — Additional file 6: Figure S6. CIRP/PHD3/HIF-1α axis aggravated apoptosis of HK-2 through the mitochondrial pathway and death receptor pathway. A Western blot analyses of extramitochondrial cytochrome c, total Apaf-1, caspase 9 and cleaved caspase 9, RPA2, Bax, Bcl-2, AIF, FADD, caspase 8 and cleaved caspase 8, caspase 3 and cleaved caspase 3 of HK-2 in the control, OGD, OGD + siNC, OGD + siCIRP, OGD + siPHD3 groups. B The ratio of extramitochondrial cytochrome c, Apaf-1 and cleaved caspase 9/caspase 9 by western blot analysis. C The ratio of RPA2, Bax, Bcl-2, AIF to β-actin by western blot analysis. D The ratio of FADD/β-actin and cleaved caspase 8/caspase8, cleaved caspase 3/caspase 3 by western blot analysis. Statistical significance was examined by one-way analysis of variance (ANOVA) followed by the Tukey test. *P < 0.05, **P < 0.01, ***P < 0.001. [file 10020_2023_655_MOESM6_ESM.jpg]

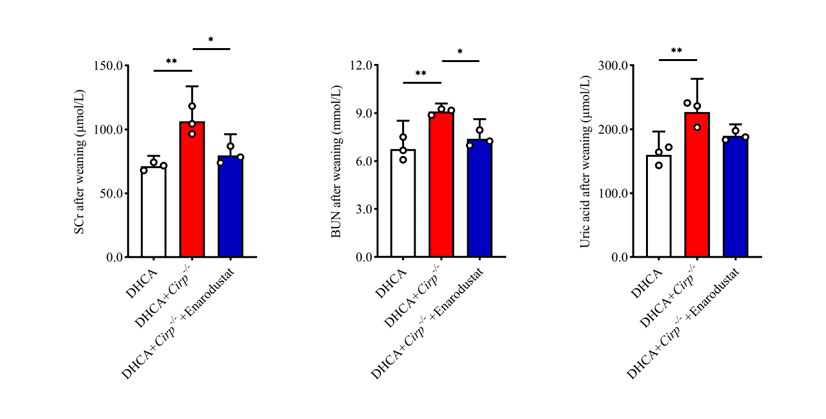

Supplement: Supplementary file 7 — Additional file 7: Figure S7. Biochemical index measurement of rat renal function. SCr, serum creatinine. BUN, blood urea nitrogen. DHCA, deep hypothermic circulatory arrest. [file 10020_2023_655_MOESM7_ESM.jpg]
