# Supplementary material for: CIRP attenuates acute kidney injury after hypothermic cardiovascular surgery by inhibiting PHD3/HIF-1α-mediated ROS-TGF-β1/p38 MAPK activation and mitochondrial apoptotic pathways
Source: Mol Med. 2023 May 1;29:61. doi: 10.1186/s10020-023-00655-0 (PMC10152741; doi:10.1186/s10020-023-00655-0)
Supplement: Supplementary file 8 — Additional file 8: Table S1. Sequences of primers used for qRT–PCR. Table S2. Primary and secondary antibodies. Table S3. Perioperative physiological index monitoring and blood-gas analysis. [file 10020_2023_655_MOESM8_ESM.doc]

| **Supplementary Table 1. Sequences of primers used for qRT–PCR** | | |
| --- | --- | --- |
| **Target gene** | **Forward (5' to 3')** | **Reverse (5' to 3')** |
| CIRP (rat) | AGTCCCGGAGTGGTGGTTA | AGGACGATCTGGACGCAGAG |
| PHD3 (rat) | TCAACTTCCTCCTGTCCCTCATCG | TTGGACCGCTCCTTGACATAGTATTTG |
| HIF-1α (rat) | TCACAAATCAGCACCAAGCAC | AAGGGGAAAGAACAAAACACG |
| KIM-1 (rat) | ATAGTGGTCTGTATTGTTGCCGAGTG | TGTGGTTGTGGGTCTTGTAGTTGTG |
| NGAL (rat) | AGCGAATGCGGTCCAGAAAGAAAG | CGAGGATGGAAGTGACGTTGTAGC |
| Cystatin C (rat) | GACTAACTGTCCTTTCCACGACCAG | CTTTCCAGGGCACGCTGTAGATC |
| β-actin (rat) | AGGCCAACCGTGAAAAGATG | ACCAGAGGCATACAGGGACAA |
| CIRP (human) | CTCCAGAGACTACTATAGCAGC | CATAACTGTCTCTGTAGGACCC |
| PHD3 (human) | GATGCCAAGCTACATGGTGGGATC | CTGCACTTCGTGTGGGTTCCTAC |
| HIF-1α (human) | CCATTAGAAAGCAGTTCCGCAAGC | GTGGTAGTGGTGGCATTAGCAGTAG |
| β-actin (human) | CTGAGAGGGAAATCGTGCGTGAC | AGGAAGAGGATGCGGCAGTGG |

| **Supplementary Table 2. Primary and secondary antibodies** | | | | |
| --- | --- | --- | --- | --- |
| **Antibody** | **Vendor** | **Catalog#** | **Dilution** | **Applications** |
| Rabbit anti-AIF antibody | Abcam | ab32516 | 1/1000 | WB |
| Rabbit anti-Apaf-1 antibody | Cell Signaling Technology | D7G4 | 1/1000 | WB |
| Rabbit anti-Bax antibody | Abcam | ab32503 | 1/1000 | WB |
| Mouse anti-Bcl-2 antibody | Santa Cruz | sc-7382 | 1/200 | WB |
| Rabbit anti-Caspase 3 antibody | Abcam | ab184787 | 1/2000, 1/1000 | WB, IHC-P |
| Rabbit anti-Cleaved Caspase 3 antibody | Cell Signaling Technology | Asp175 | 1/1000, 1/1000 | WB, IHC-P |
| Mouse anti-Caspase 8 antibody | Santa Cruz | sc-81656 | 1/200 | WB |
| Rabbit anti-Caspase 9 antibody | Abcam | ab184786 | 1/1000 | WB |
| Rabbit anti-Caspase 9 antibody | Abcam | ab202068 | 1/2000 | WB |
| Rabbit anti-CIRP antibody | Abcam | ab191885 | 1/1000, 1/1000 | WB, ICC |
| Rabbit anti-CIRP antibody | Abcam | ab246510 | 1/1000 | WB |
| Rabbit anti-CIRP antibody | Proteintech | 10209-2-AP | 1/50 | IF |
| Rabbit anti-Cytochrome c antibody | Cell Signaling Technology | D18C7 | 1/1000, 1/100 | WB, IHC-P |
| Rabbit anti-FADD antibody | Proteintech | 14906-1-AP | 1/1000, 1/50 | WB |
| Mouse anti-HIF-1α antibody | Abcam | ab1 | 1/1000, 1/100, 1/100 | WB, ICC, IF |
| Rabbit anti-HIF-1α antibody | Abcam | ab179483 | 1/1000, 1/500, 1/500 | WB, ICC, IF |
| Rabbit anti-p38 MAPK antibody | Cell Signaling Technology | D13E1 | 1/1000, 1/400 | WB, IHC-P |
| Rabbit anti-phospho-p38 MAPK antibody | Cell Signaling Technology | D3F9 | 1/1000, 1/800 | WB, IHC-P |
| Rabbit anti-PHD3 antibody | Abcam | ab184714 | 1/2000, 1/250, 1/250 | WB, IHC-P, ICC |
| Rabbit anti-RPA3 antibody | Abcam | ab76420 | 1/1000 | WB |
| Rabbit anti-TGF-β1 antibody | Abcam | ab215715 | 1/1000, 1/500 | WB, IHC-P |
| Rabbit anti-TRX antibody | Abcam | ab109385 | 1/10000 | WB |
| Mouse anti-β-actin antibody | Proteintech | 66009-1-Ig | 1/10000 | WB |
| Goat anti-mouse IgG H&L (HRP) | ZSGB-BIO | ZB-2305 | 1/5000 | WB |
| Goat anti-rabbit IgG H&L (HRP) | ZSGB-BIO | ZB-2301 | 1/5000 | WB |
| Goat anti-mouse IgG H&L (HRP)(Alexa Fluor® 647) | Abcam | ab150115 | 1/500 | IF |
| Goat anti-rabbit IgG H&L (HRP)(Alexa Fluor® 488) | Abcam | ab150077 | 1/500, 1/500 | IF, ICC |
| Goat anti-rabbit IgG H&L (HRP) | ZSGB-BIO | PV-6001 | 1/500 | IHC-P |

| **Supplementary Table 3. Perioperative physiological index monitoring and blood-gas analysis** | | | | | | |
| --- | --- | --- | --- | --- | --- | --- |
| **Variables** | **Time points** | **Sham** | **DHCA** | **DHCA+CIRP^-/-^** | ***P* value** |  |
| MAP, mmHg | T0 | 99.80 ± 11.17 | 99.20 ± 8.90 | 99.20 ± 9.83 | 0.994 |  |
|  | T1 | 95.60 ± 9.97 | 90.60 ± 5.51 | 88.20 ± 5.12 | 0.291 |  |
|  | T2 | 96.20 ± 10.28 | 72.80 ± 5.07 | 68.80 ± 3.42 | <0.001 |  |
|  | T3 | 94.40 ± 8.96 | 55.60 ± 5.68 | 49.80 ± 3.35 | <0.001 |  |
|  | T4 | 91.00 ± 8.25 | 60.40 ± 4.83 | 56.40 ± 3.98 | <0.001 |  |
| HR, bpm | T0 | 287.40 ± 24.48 | 274.60 ± 13.39 | 272.00 ± 10.84 | 0.353 |  |
|  | T1 | 288.00 ± 13.02 | 228.60 ± 8.50 | 234.40 ± 6.23 | <0.001 |  |
|  | T2 | 290.80 ± 9.99 | 68.60 ± 8.59 | 63.40 ± 5.27 | <0.001 |  |
|  | T3 | 286.00 ± 4.64 | 103.60 ± 13.47 | 94.60 ± 15.29 | <0.001 |  |
|  | T4 | 286.20 ± 6.80 | 240.20 ± 11.17 | 242.00 ± 17.31 | <0.001 |  |
| Hb, g/L | T0 | 137.20 ± 7.69 | 136.20 ± 5.81 | 137.30 ± 7.33 | 0.967 |  |
|  | T1 | 133.20 ± 6.83 | 78.80 ± 6.46 | 78.20 ± 5.76 | <0.001 |  |
|  | T2 | 131.80 ± 6.98 | 76.20 ± 5.68 | 77.60 ± 4.93 | <0.001 |  |
|  | T3 | 130.80 ± 6.26 | 75.40 ± 5.18 | 73.80 ± 3.27 | <0.001 |  |
|  | T4 | 130.20 ± 6.54 | 70.40 ± 5.68 | 67.00 ± 2.74 | <0.001 |  |
| Hct, % | T0 | 46.20 ± 2.59 | 45.00 ± 2.65 | 45.60 ± 2.30 | 0.758 |  |
|  | T1 | 44.00 ± 2.35 | 26.20 ± 2.39 | 25.80 ± 1.64 | <0.001 |  |
|  | T2 | 43.60 ± 2.97 | 25.20 ± 1.10 | 24.60 ± 2.07 | <0.001 |  |
|  | T3 | 43.40 ± 1.67 | 24.60 ± 1.52 | 24.00 ± 1.23 | <0.001 |  |
|  | T4 | 43.20 ± 1.64 | 23.60 ± 1.95 | 23.60 ± 1.52 | <0.001 |  |
| pH | T0 | 7.40 ± 0.02 | 7.39 ± 0.04 | 7.38 ± 0.03 | 0.808 |  |
|  | T1 | 7.44 ± 0.03 | 7.48 ± 0.03 | 7.47 ± 0.03 | 0.082 |  |
|  | T2 | 7.45 ± 0.02 | 7.51 ± 0.03 | 7.51 ± 0.02 | 0.003 |  |
|  | T3 | 7.48 ± 0.03 | 7.38 ± 0.08 | 7.36 ± 0.06 | 0.017 |  |
|  | T4 | 7.42 ± 0.02 | 7.41 ± 0.03 | 7.44 ± 0.02 | 0.142 |  |
| PaO_2_, mmHg | T0 | 280.38 ± 27.35 | 269.98 ± 30.84 | 282.68 ± 26.42 | 0.731 |  |
|  | T1 | 285.26 ± 46.84 | 261.78 ± 30.14 | 265.02 ± 22.02 | 0.526 |  |
|  | T2 | 298.22 ± 26.88 | 272.68 ± 10.96 | 256.98 ± 49.80 | 0.184 |  |
|  | T3 | 302.86 ± 36.23 | 287.08 ± 18.60 | 284.14 ± 14.79 | 0.468 |  |
|  | T4 | 307.08 ± 48.69 | 290.32 ± 13.22 | 278.40 ± 15.88 | 0.360 |  |
| PaCO_2_, mmHg | T0 | 38.24 ± 5.18 | 39.00 ± 3.10 | 40.40 ± 3.79 | 0.708 |  |
|  | T1 | 34.32 ± 3.88 | 32.56 ± 4.16 | 32.52 ± 4.02 | 0.727 |  |
|  | T2 | 33.40 ± 5.23 | 31.66 ± 4.07 | 30.92 ± 4.50 | 0.692 |  |
|  | T3 | 36.06 ± 5.03 | 32.04 ± 5.71 | 31.36 ± 5.76 | 0.376 |  |
|  | T4 | 39.66 ± 3.16 | 36.54 ± 4.60 | 35.90 ± 3.46 | 0.283 |  |
| SaO_2_, mmHg | T0 | 99.18 ± 0.65 | 98.62 ± 0.83 | 99.04 ± 0.92 | 0.540 |  |
|  | T1 | 99.40 ± 0.39 | 99.08 ± 0.26 | 99.20 ± 0.27 | 0.298 |  |
|  | T2 | 99.22 ± 0.28 | 99.40 ± 0.58 | 99.26 ± 0.18 | 0.748 |  |
|  | T3 | 99.34 ± 0.27 | 99.56 ± 0.24 | 99.64 ± 0.11 | 0.123 |  |
|  | T4 | 99.12 ± 0.79 | 99.54 ± 0.11 | 99.62 ± 0.18 | 0.236 |  |
| Lac, mmol/L | T0 | 0.96 ± 0.47 | 1.36 ± 0.54 | 1.64 ± 0.80 | 0.256 |  |
|  | T1 | 1.42 ± 0.57 | 2.00 ± 0.58 | 2.08 ± 0.52 | 0.166 |  |
|  | T2 | 1.60 ± 0.46 | 2.02 ± 0.38 | 2.22 ± 0.55 | 0.147 |  |
|  | T3 | 1.76 ± 0.34 | 8.60 ± 1.70 | 9.58 ± 2.19 | <0.001 |  |
|  | T4 | 2.04 ± 0.29 | 9.04 ± 1.24 | 10.28 ± 1.91 | <0.001 |  |
| Values are presented as mean ± standard deviation (SD). MAP, mean arterial pressure. HR, heart rate. Hb, hemoglobin. Hct, hematocrit. PaO_2_, partial arterial oxygen pressure. PaCO_2_, arterial partial pressure of carbon dioxide. SaO_2_, arterial oxygen saturation. Lac, lactic acid. T0, before CPB initiation; T1, 5 min after cooling; T2, 30 min after cooling; T3, 5 min after rewarming; T4, at the weaning of CPB. | | | | | |  |
|  |  |  |  |  |  |  |
|  |  |  |  |  |  |  |
|  |  |  |  |  |  |  |
